# Complementary Eigen–Zundel Interpretation Reconciles Thermodynamics and Spectroscopy of Excess Protons in Aqueous HF Solutions

**DOI:** 10.1002/anie.3996002

**Published:** 2026-09-30

**Authors:** Louis Lehmann, Florian N. Brünig, Jonathan Scherlitzki, Morten Lehmann, Martin Kaupp, Beate Paulus, Roland R. Netz

**Affiliations:** ^1^ Department of Physics Freie Universität Berlin Berlin Germany; ^2^ Department of Physics and Materials Science University of Luxembourg Luxembourg City Luxembourg; ^3^ Department of Physical and Theoretical Chemistry Freie Universität Berlin Berlin Germany; ^4^ Institute of Chemistry Technische Universität Berlin Berlin Germany

**Keywords:** ab‐initio MD, Eigen–Zundel, hydrofluoric acid, proton transfer, spectroscopy

## Abstract

Aqueous solutions of HF and HCl behave very differently at intermediate concentrations: HCl dissociates completely, whereas HF only partially dissociates and forms bifluoride (${\mathrm{HF}}_{2}^{-}$). This should lead to markedly different excess‐proton spectra in HF and HCl solutions, in contrast to experimental reports. Using ab initio molecular dynamics, we show that in HF the proton is not firmly bound to $F^{-}$, as suggested by textbook chemistry, but dynamically shared with a hydrating water molecule. This is rationalized by a modified Eigen‐picture description which also explains the formation of ${\mathrm{HF}}_{2}^{-}$. The similar vibrational spectra of HF and HCl solutions are explained by a complementary Zundel picture in terms of almost identical excess proton transfer free‐energy profiles for HF and HCl. These results reconcile thermodynamic and spectroscopic observations and provide a unified microscopic picture of excess protons in aqueous solution.

## Graphical abstract

Aqueous solutions of HF and HCl show contrasting dissociation in water, yet exhibit nearly identical excess‐proton spectra. This puzzling observation is reconciled by a complementary Eigen–Zundel interpretation.

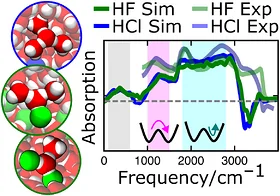

## 1 Introduction

Understanding the behavior of excess protons in aqueous solution is of fundamental importance in many areas of chemistry and biology, such as proton‐coupled electron transfer processes [1, 2], electrocatalytic reactions [3], enzymatic reactions [4], membrane proton diffusion [5, 6], and fuel cells [7, 8]. The thermodynamic properties of an acid in aqueous solution are well described by the acid dissociation[Supplementary-material anie74941-supl-0001] reaction

(1) $$\mathrm{HA}⇌A^{-}+H^{+}\;,$$

where HA is the acid molecule, $A^{-}$ its conjugated base, and $H^{+}$ is the excess proton [9]. Strong acids such as hydrochloric acid (HCl) ($A^{-}={\mathrm{Cl}}^{-}$, $pK_{a}=-7.0$) dissociate almost completely in water, while weak acids such as hydrofluoric acid (HF) ($A^{-}=F^{-}$, $pK_{a}=3.2$) only partially [9]. Measurements of electromotive force [10, 11], conductivity [12, 13], the Hammett acidity function [14, 15], and calorimetry [16] indicate that at moderate HF concentrations $F^{-}$ is converted into bifluoride (${\mathrm{HF}}_{2}^{-}$) according to the following reaction:

(2) $$\begin{array}{c} \mathrm{HF}+F^{-}⇌{\mathrm{HF}}_{2}^{-}, \end{array}$$

which increases the activity of $H^{+}$ according to Le Chatelier's principle. It has been known for almost a century that acids with vastly different dissociation behavior exhibit similar IR spectra [17]. Solutions of HF and HCl in particular were investigated by Giguère et al. [18, 19, 20] who found no spectral evidence for ${\mathrm{HF}}_{2}^{-}$. Instead, they proposed that the observed spectra can be explained by a Zundel‐like $H_{3}\mathrm{OF}$ configuration in which the proton is shared between water and fluoride.

Previous ab‐initio molecular dynamics (AIMD) simulations of HF solutions [21, 22, 23, 24, 25] identified a significant population of $H_{3}\mathrm{OF}$ configurations, while no formation of ${\mathrm{HF}}_{2}^{-}$ ions was observed at moderate concentrations, consistent with Giguère's interpretation of experimental spectra. Simon and Klein [23] observed spontaneous ${\mathrm{HF}}_{2}^{-}$ formation in AIMD simulations of an equimolar HF–water mixture, demonstrating that ${\mathrm{HF}}_{2}^{-}$ formation is accessible on AIMD timescales, but not favored at moderate concentrations, in apparent contradiction with thermodynamic predictions. Rather than invoking ${\mathrm{HF}}_{2}^{-}$ formation, Sillanpää et al. [22] proposed that the increasing acidity at higher concentrations can be partly attributed to enhanced fluoride stabilization arising from the increasing ionic strength of the solution. This interpretation, however, is not fully consistent with Debye–Hückel theory, which predicts that increased ionic strength stabilizes all charged species and thus lowers the activity of excess protons as well.

In strong acids, excess‐proton hydrates are typically interpreted as Zundel ($H_{5}O_{2}^{+}$) or Eigen ($H_{9}O_{4}^{+}$) cations, representing limiting cases of proton sharing between two water molecules or proton localization on a hydronium ion stabilized by three hydrogen‐bonded water molecules [26, 27, 28, 29, 30, 31, 32, 33, 34, 35, 36, 37, 38]. The minimal motif that explains spectral signatures of excess protons in strong acids is Zundel‐like, with an excess proton shared between two water molecules [34, 39], which was utilized by Brünig et al. to identify spectral signatures of proton transfer [39]. However, structurally excess‐proton hydrates in strong acids more closely resemble distorted Eigen‐cations, whereas Zundel‐like configurations are commonly interpreted as transition states during proton transfer [27, 33, 40, 41, 42]. At higher HCl concentrations, chloride can replace a water molecule in the distorted Eigen cation [32, 43], creating an entropic barrier for proton transport [32]. Excess protons have been experimentally extensively characterized by infrared (IR) spectroscopy [19, 28, 30, 44, 45, 46, 47, 48, 49, 50, 51]. Multistate empirical valence bond simulations [27, 33, 45], AIMD [39, 52, 53], and machine‐learning molecular dynamics [35, 37, 54, 55] have reproduced characteristic excess‐proton IR signatures in strong acids, in some cases even without including the conjugate base [35, 54, 55]. AIMD studies of HF report substantially different IR broadband signatures for chemical species appearing in HF solutions [22, 52]. Other studies focused on cryogenic temperatures and found pronounced spectral differences between the hydrohalic acids and proton‐sharing intermediates [24, 25].

Thermodynamics predicts markedly different chemical speciation in aqueous HF and HCl, whereas their experimental spectra are very similar, which is puzzling. We analyze AIMD simulations by an extended Eigen framework, which yields chemical speciation consistent with thermodynamic expectations, including bifluoride formation. Spectral proton transfer signatures are best understood in a complementary Zundel picture, by which we explain why the proton‐transfer bands of HF and HCl solutions are very similar under ambient conditions and typical experimental concentrations (7.5 mol/kg), even though the majority of excess protons in HF are in direct contact with fluoride, while in HCl they are mainly shared between water molecules. The puzzling similarity arises because electrostatic screening renders the free‐energy landscapes within the Zundel configurations $H_{5}O_{2}^{+}$ (dominant in HCl) and $H_{3}\mathrm{OF}$ (dominant in HF) nearly indistinguishable, leading to similar proton‐transfer dynamics and thereby similar IR signatures of proton transfer. Throughout, we use the Eigen and Zundel pictures as complementary views of the excess proton: the Eigen picture describes a central hydronium ion hydrogen‐bonded to three proton acceptors, whereas the Zundel picture focuses on proton sharing of the hydronium's most weakly bound proton with a single proton acceptor.

## 2 Results and Discussion

### 2.1 IR Spectra and Chemical Speciation

We begin our analysis by comparing the solvation structure of excess protons in 7.5 mol/kg HF and HCl solutions at ambient conditions using AIMD simulations. Simulation snapshots are shown in Figure 1a, along with a zoom into the prevalent excess‐proton hydration structures, which are mainly coordinated by fluoride and water in HF solutions, and mainly by water in HCl solutions.

**FIGURE 1:**
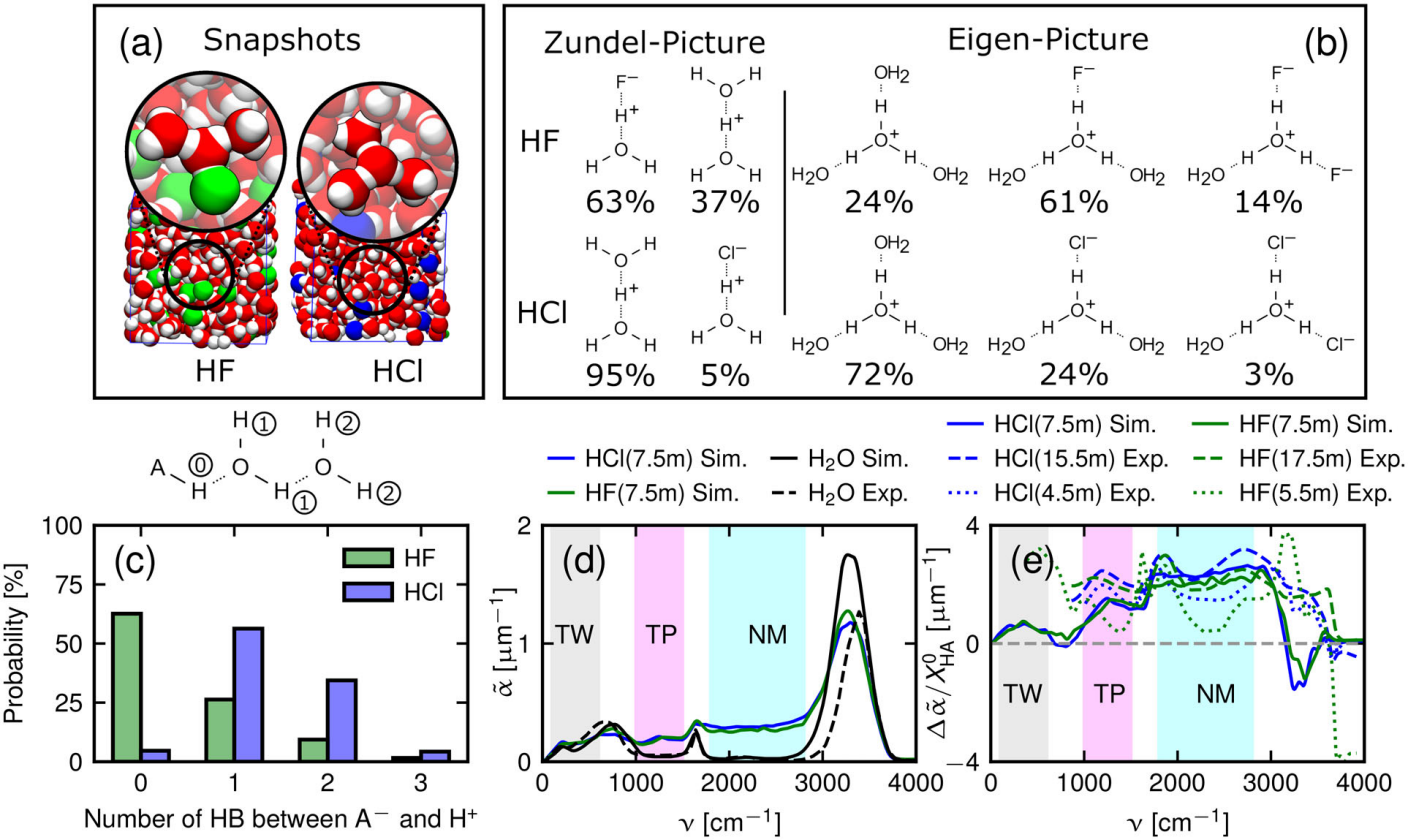
Analysis of excess‐proton solvation, and IR spectra of 7.5 mol/kg HF and HCl solutions. (a) Simulation snapshots of systems containing 224 water molecules and 30 acid molecules, with HF shown on the left and HCl on the right, each including a zoomed view on representative Eigen structures. (b) Chemical structures and probability distribution of excess proton solvation in the Zundel‐picture and Eigen‐picture for the two acid solutions. (c) Probability of finding an excess proton ($H^{+}$) and its conjugate base ($A^{-}$) in HF and HCl separated by a minimal number of hydrogen bonds, as indicated by the circled numbers in the sketch above the panel. (d) Simulated absorption coefficients $\tilde{\alpha }\left(\omega \right)$ of pure water, and HF and HCl solutions. The experimental absorption coefficient of pure water [56] is shown for comparison. (e) Rescaled difference spectra, as defined in Equation (3), for the simulated acid solutions compared with experimental difference spectra extracted from the literature: HF (5.5 m) [44], HCl (4.5 m) [47], HCl (15.5 m) [18], HF (17.5 m) [18]. The colored frequency ranges correspond to the transfer‐waiting (TW, gray), transfer‐path (TP, magenta), and normal‐mode (NM, cyan) regions [39]. Concentrations are given in units of molality m=mol/kg.

An analysis of proton solvation beyond the Zundel–Eigen picture is shown in Figure 1c, which reports the probability of finding the excess proton and its conjugate base separated by the minimal number of hydrogen bonds (i.e., along the shortest hydrogen‐bond path), as illustrated schematically above the panel. In HF, fluoride most frequently remains in direct contact with the excess proton (0 hydrogen bonds). Each additional hydrogen bond roughly halves this probability, and separations beyond two hydrogen bonds are rare, limiting the participation of excess protons in electrode reactions [10, 11] and conductivity [12, 13]. This is different for HCl, where excess protons are effectively repelled from chloride ions and are therefore most commonly separated by one or two hydrogen bonds. This difference is also reflected in the theoretically determined excess‐proton mobility: relative to water, excess protons diffuse ∼3.7 times faster in HCl but only ∼1.3 times faster in HF, as shown in the Supporting information.

A complementary Zundel–Eigen interpretation is applied to HF and HCl solutions and presented in Figure 1b. In the Zundel‐picture the excess proton is shared between two proton acceptors, which can be either water or the conjugate base. In HF solutions, $H_{3}\mathrm{OF}$ configurations dominate (63%), whereas $H_{5}O_{2}^{+}$ accounts for only 37%. In contrast, HCl solutions are overwhelmingly characterized by $H_{5}O_{2}^{+}$ structures (95%), with $H_{3}\mathrm{OCl}$ being only marginally found (5%). These differences reflect the distinct proton solvation preferences in weak and strong acids: fluoride participates directly in proton sharing in a substantial fraction of configurations, whereas chloride plays only a minor role in the corresponding HCl solutions.

In the Eigen picture the excess proton is distributed over four proton acceptors, illustrated by a central hydronium ion ($H_{3}O^{+}$) hydrogen‐bonded to three surrounding molecules, which may be water or the conjugate base. The localization of the positive charge on the hydronium ion in Figure 1b reflects the conventional picture of a hydrogen‐bond‐stabilized $H_{3}O^{+}$ ion, whereas in reality these hydrogen bonds can have significant covalent character, especially in the case of fluoride. In HCl solutions, the conventional Eigen‐cation $H_{9}O_{4}^{+}$ is the dominant configuration (72%), with other Eigen configurations such as $H_{7}O_{3}\mathrm{Cl}$ (24%) and $H_{5}O_{2}{\mathrm{Cl}}_{2}^{-}$ (3%) being less likely, as shown in Figure 1b. In HF solutions, the excess proton is found in the Eigen cation $H_{9}O_{4}^{+}$ only in 24% of cases. More commonly, it appears in $H_{7}O_{3}F$ structures (61%), while 14% of excess protons are coordinated by two water molecules and two fluoride ions, forming $H_{5}O_{2}F_{2}^{-}$ configurations. Further, roughly 1% of excess protons are coordinated by three fluoride ions (not shown), which leaves only 8% of fluoride ions uncoordinated by excess protons. Because water is present in large excess, adding or removing water molecules from these structures does not change the underlying chemical equilibrium. $H_{9}O_{4}^{+}$, $H_{7}O_{3}F$, and $H_{5}O_{2}F_{2}^{-}$ can therefore be interpreted as hydrates of $H^{+}$, HF, and ${\mathrm{HF}}_{2}^{-}$, respectively. Thermodynamic analysis based on electromotive‐force measurements and activity‐coefficient estimates predicts $H^{+}$ and $F^{-}$ populations of 14% and 0.1% in HF solutions at 6 mol/kg [11]. Using charge and fluorine balance (see Supporting Information), this corresponds to 14%, 72%, and 14% for $H^{+}$, HF, and ${\mathrm{HF}}_{2}^{-}$. These values compare rather well with 24%, 61%, and 14% obtained here at 7.5 mol/kg. Overall, the chemical speciation is consistent, especially given the experimental uncertainties and the modeling approximations discussed in the Supporting information, namely, the choice of DFT functional [33, 57, 58, 59] and the neglect of nuclear quantum effects [53, 55].

We present the simulated absorption coefficients $\tilde{\alpha }\left(\omega \right)$ of HF and HCl solutions and water in Figure 1d, where $\tilde{\alpha }\left(\omega \right)$ denotes the inverse distance over which the intensity of incident light decays to 1/e due to absorption [60]. The extraction of $\tilde{\alpha }\left(\omega \right)$ from AIMD is described in the Supporting information and quantitatively tested by comparison between simulated (solid black) and experimental (dashed black) absorption coefficients of pure water [56] in Figure 1d. The agreement is good, with a slight overestimation in the OH‐stretch region (3000–3700 ${\mathrm{cm}}^{-1}$), consistent with previous results [61]. We observe that the absorption coefficients of the HF and HCl solutions are significantly different from pure water. Vibrational signatures of excess protons are most clearly identified in rescaled difference spectra,

(3) $$\begin{array}{cc} \frac{\Delta \tilde{\alpha }\left(\omega \right)}{X_{\mathrm{HA}}^{0}} & =\frac{\tilde{\alpha }\left(\omega \right)-X_{H_{2}O}{\tilde{\alpha }}_{H_{2}O}\left(\omega \right)}{X_{\mathrm{HA}}^{0}}, \end{array}$$

where $X_{\mathrm{HA}}^{0}$ is the total mole fraction of acid added to the solution, and $X_{H_{2}O}$ is the mole fraction of water molecules excluding the water molecules that form the central hydronium ions in the Eigen picture. ${\tilde{\alpha }}_{H_{2}O}\left(\omega \right)$ denotes the absorption coefficient of pure water. As shown in Ref.  [39], where a similar construction of the difference spectra was employed, spectral signatures defined in this way in HCl solutions vary only weakly with concentration. In the simulated and experimental rescaled difference spectra shown in Figure 1e, the transfer waiting (TW), transfer path (TP) and normal mode (NM) bands are clearly visible [39].

Here, the TW band reflects the proton‐transfer reaction frequency or waiting time, the TP band arises from the proton TP time, and the NM band reflects vibrational oscillations of the proton bound to a base [39] and overlaps with the inverse timescale of the special pair dance [41], which is reported to be 15.7 fs in Ref. [33], corresponding to a frequency of 2125 ${\mathrm{cm}}^{-1}$. Due to the fundamentally different distributions of Zundel‐like structures in HF and HCl solutions presented in Figure 1b, one would expect significantly different spectra. Rather surprisingly, however, we find that the proton‐transfer signatures (TW and TP) are nearly indistinguishable between the two acids, while the NM region exhibits small differences. A comparison with experimental difference spectra of HF and HCl solutions in Figure 1e, which we constructed from absorption spectra reported in the literature [18, 44, 47] as described in Supporting information, shows that HF solutions display pronounced proton‐transfer signatures that closely resemble those observed in HCl solutions, in agreement with our theoretical predictions.

In summary, applying the Eigen picture to weak acids yields chemical speciation consistent with thermodynamic predictions, including the debated bifluoride ion. For analyzing spectroscopic signatures of proton transfer, however, the Zundel picture is more useful, since it allows to link the dynamics of the proton‐transfer reaction coordinate to IR spectra [39]. Given the significant fraction of excess protons in HF coordinated by two water molecules, one might expect similarities between the spectra of HF and HCl solutions. However, the almost indistinguishable spectral proton‐transfer signatures are puzzling given that the majority of excess protons are in direct contact with fluoride in HF.

### 2.2 Free Energy Landscapes of Proton Transfer within $H_{5}O_{2}^{+}$ and $H_{3}\mathrm{OF}$

We analyze the free energy of the excess proton as a function of the coordinates R and d, illustrated in Figure 2a. Here, R denotes the distance between the heavy atoms and the proton‐transfer coordinate d is defined as the projection of the excess proton position onto the heavy‐atom connecting axis (O–O, F–O, or Cl–O).

**FIGURE 2:**
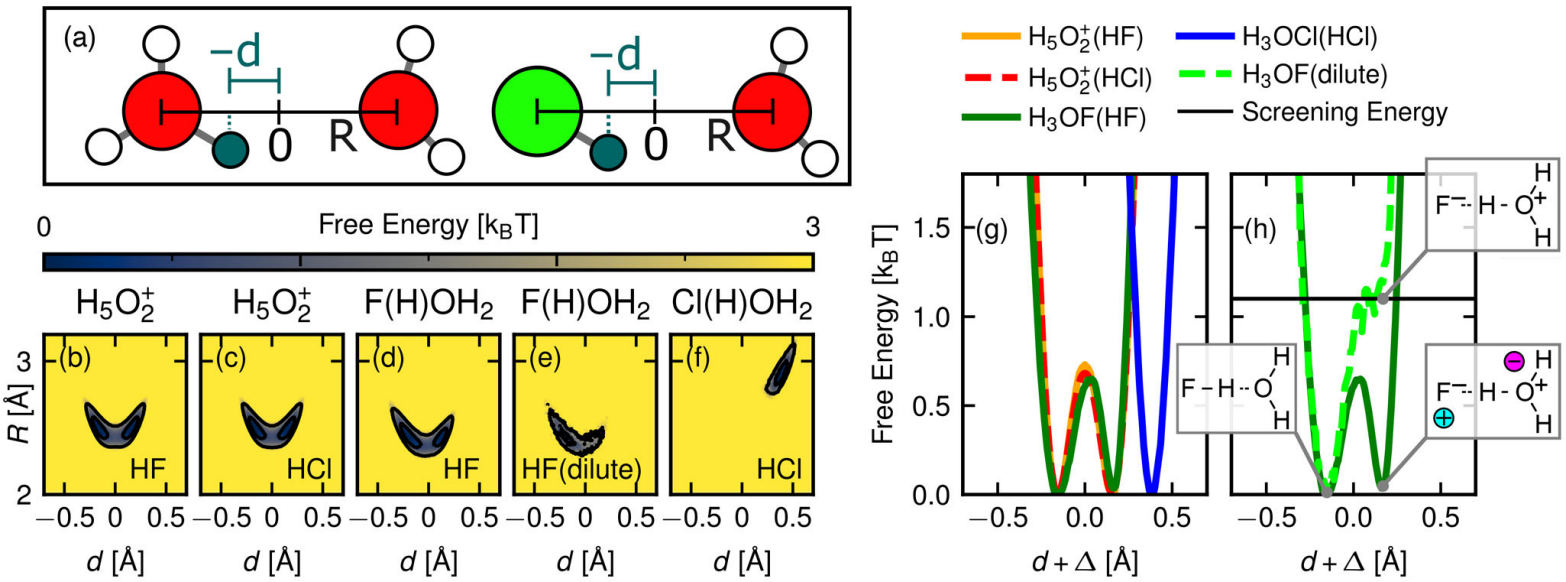
Analysis of the excess‐proton free‐energy landscapes in terms of the proton‐transfer coordinate d and the heavy‐atom‐distance R, as schematically illustrated in (a): the coordinate d is the projection of the excess‐proton position onto the heavy‐atom connecting vector axis (O–O, F–O, or Cl–O) of length R. The excess proton is colored in dark turquoise and in all cases, negative d correspond to the proton being closer to the left heavy atom. (b–f) Two‐dimensional free‐energy surfaces of the excess proton for the Zundel configuration: $H_{5}O_{2}^{+}$ in HF, $H_{5}O_{2}^{+}$ in HCl, $H_{3}\mathrm{OF}$ in HF, $H_{3}\mathrm{OF}$ in dilute HF, and $H_{3}\mathrm{OCl}$ in HCl, respectively. (g) One‐dimensional free‐energy profiles along the proton‐transfer coordinate d, for the Zundel configuration in concentrated HF and HCl solutions. (h) Same as in (g), but only for $H_{3}\mathrm{OF}$ in concentrated and in dilute HF to highlight the effect of screening stabilization of the ion pair $F^{-}\cdots H_{3}O^{+}$ in HF. The free‐energy shift of the ion pair $F^{-}\cdots H_{3}O^{+}$ of 1.1 $k_{B}T$ between the two concentrations is indicated by the horizontal line. The mechanism underlying the energetic similarity between $H_{5}O_{2}^{+}$ and $H_{3}\mathrm{OF}$ in HF is illustrated schematically by the chemical structures and surrounding ions shown in the insets of (h). To facilitate comparison with $H_{5}O_{2}^{+}$, the d coordinate of $H_{3}\mathrm{OF}$ is shifted by Δ=0.04Å in (g) and (h).

As can be seen by comparison of Figure 2b‐d, the two‐dimensional free‐energy surfaces of $H_{5}O_{2}^{+}$ in HF, $H_{5}O_{2}^{+}$ in HCl and $H_{3}\mathrm{OF}$ in HF are very similar, with the most notable difference being a slight shift of the excess proton toward fluoride (i.e. to the left) for large heavy‐atom distances R in $H_{3}\mathrm{OF}$. All three free‐energy surfaces exhibit the characteristic double‐well shape for large heavy‐atom distances R and a single‐well shape for small R, which is typical for Zundel configurations and is qualitatively consistent with previous works [22, 23, 26, 39, 62]. The striking similarity between the free‐energy surfaces of $H_{5}O_{2}^{+}$ (Figures 2b,c) and $H_{3}\mathrm{OF}$ in HF (Figure 2d) explains the nearly identical proton‐transfer signatures observed in the IR spectra of HF and HCl solutions.

In the $H_{3}\mathrm{OCl}$ configuration the proton is strongly localized toward the water molecule, as shown in Figure 2f, which is a consequence of the negligible proton affinity of chloride. In order to understand the underlying mechanism for the energetic similarity between $H_{5}O_{2}^{+}$ and $H_{3}\mathrm{OF}$ in HF, we additionally simulated a single HF molecule in water using AIMD and computed the free‐energy surface of $H_{3}\mathrm{OF}$ in dilute HF, shown in Figure 2e. As can be seen by comparison of Figure 2d,e, the proton in $H_{3}\mathrm{OF}$ in dilute HF is localized slightly more toward the fluoride ion, while in concentrated HF it is almost equally shared between the water molecule and the fluoride ion, with a free‐energy landscape comparable to that of $H_{5}O_{2}^{+}$.

To enable a more quantitative comparison between the different Zundel configurations, we present one‐dimensional free‐energy profiles along the proton‐transfer reaction coordinate d, obtained by integrating the two‐dimensional probability distribution over R, which we show in Figure 2g,h. Approximately, the reaction coordinate d describes the proton transfer reaction according to the following reaction:

(4) $$\begin{array}{c} \mathrm{AH}\cdots {\mathrm{OH}}_{2}⇌A^{-}\cdots H_{3}O^{+}, \end{array}$$

with $A\in \{H_{2}O^{+},F,\mathrm{Cl}\}$, where the dots indicate hydrogen bonds. The state $\mathrm{AH}\cdots {\mathrm{OH}}_{2}$ (d<0) corresponds to the proton bound to $A^{-}$, and $A^{-}\cdots H_{3}O^{+}$ (d>0) is the configuration where the excess proton is localized on the water molecule. As can be seen in Figure 2g, the Δ‐shifted one‐dimensional free‐energy profiles of $H_{5}O_{2}^{+}$ in HF (solid orange), $H_{5}O_{2}^{+}$ in HCl (dashed red) and $H_{3}\mathrm{OF}$ (solid dark green) in HF are almost indistinguishable, with barriers around 0.7 $k_{B}T$. In contrast, the free‐energy profile of $H_{3}\mathrm{OCl}$ in HCl (solid blue) is highly asymmetric, exhibiting a single well localized toward the water molecule, consistent with the negligible proton affinity of chloride. The free‐energy profiles of $H_{3}\mathrm{OF}$ in concentrated HF (solid dark green) and in dilute HF (dashed lime green) are shown in Figure 2h to highlight the effect of screening on the stabilization of the ion pair $F^{-}\cdots H_{3}O^{+}$. In dilute HF, $H_{3}\mathrm{OF}$ exhibits a single well localized toward the fluoride ion. In concentrated HF, however, the ion pair is stabilized by the surrounding ionic environment, resulting in a free‐energy lowering of approximately $1.1\;k_{B}T$, as indicated by the horizontal line in Figure 2h.

The free energy stabilization of two ions $F^{-}$ and $H_{3}O^{+}$ at separation R due to ionic screening is predicted by Debye–Hückel theory [63] to be

(5) $$\begin{array}{c} \Delta F=-\frac{e^{2}}{4\pi \varepsilon_{0}\varepsilon }\frac{\kappa }{1+\kappa R}=-1.5\;k_{B}T, \end{array}$$

in decent agreement with the observed free‐energy difference of $-1.1\;k_{B}T$ between the 6.5 mol/L and the dilute solution in Figure 2h. Here, e is the elementary charge, $\varepsilon_{0}$ is the vacuum permittivity, $\kappa^{-1}=\;2.4\;Å$ is the Debye length at concentration of free ions of $c=24\%\times 6.5\;\mathrm{mol}\;L^{-1}$, R=2.4Å is the mean distance between the hydronium and the fluoride ion (compare Figure 2d,e) and ε=78 is the dielectric constant of water [64]. Since the equilibrium in Equation (4) remains unchanged for $H_{5}O_{2}^{+}$ due to symmetry but shifts as a function of ionic strength for $H_{3}\mathrm{OF}$, one expects a stronger concentration dependence of proton‐transfer spectra in HF than in HCl, where it was found to be minimal [39].

## 3 Conclusion

Our analysis reconciles thermodynamic and spectroscopic properties of aqueous HF and provides a consistent picture of excess proton solvation in the presence of conjugate bases with significant proton affinity, such as $F^{-}$. An extended Eigen framework yields chemical speciation consistent with thermodynamic predictions, including the elusive bifluoride ion. Despite distinct excess‐proton structures found in HF and HCl, the proton‐transfer bands are nearly identical. This apparent contradiction is resolved by screening due to free ions, which renders the free‐energy profiles of the proton transfer coordinate within the relevant Zundel configurations nearly indistinguishable in HF and HCl, leading to convergent proton‐transfer dynamics and spectroscopic signatures. These free‐energy profiles and spectral signatures are connected through the generalized Langevin equation  [61, 65]. Our complementary Eigen–Zundel interpretation is crucial: Eigen‐like structures determine equilibrium populations, while Zundel‐like configurations encode the transition states that govern vibrational response. We expect that our findings are applicable to weak acids in general. However, we suggest that the small size of $F^{-}$ enables the formation of $H_{5}O_{2}F_{2}^{-}$, which would be sterically hindered for larger anions and therefore explains the anomalous thermodynamic behavior of HF compared to other weak acids.

## Conflicts of Interest

The authors declare no conflicts of interest.

## Supporting information


**Supporting File**: anie74941‐sup‐0001‐SuppMat.pdf.

## Data Availability

The data that support the findings of this study are available from the corresponding author upon reasonable request.
