# Supplementary material for: Complementary Eigen–Zundel Interpretation Reconciles Thermodynamics and Spectroscopy of Excess Protons in Aqueous HF Solutions
Source: Angew Chem Int Ed Engl. 2026 Sep 30;65(46):e3996002. doi: 10.1002/anie.3996002 (PMC13749355; doi:10.1002/anie.3996002)
Supplement: Supplementary file 1 [file ANIE-65-e3996002-s001.pdf]

Supplemental Information

Louis Lehmann,[1] Florian N. Brünig,[2] Jonathan Scherlitzki,[3] Morten Lehmann,[4] Martin Kaupp,[4] Beate Paulus,[3] and Roland R. Netz*[1]

[1]*Department of Physics, Freie Universität Berlin, Arnimallee 14, 14195 Berlin, Germany*
[2]*Department of Physics and Materials Science, University of Luxembourg, L-1511 Luxembourg City, Luxembourg*
[3]*Department of Physical and Theoretical Chemistry, Freie Universität Berlin, Arnimallee 22, 14195 Berlin, Germany*
[4]*Institute of Chemistry, Technische Universität Berlin, Straße des 17. Juni 115, 10623 Berlin, Germany*
(*corresponding author: rnetz@physik.fu-berlin.de)

CONTENTS

S.I. Computational Details 2

S.II. Discussion of the Employed Level of Theory and Quantum Chemistry Calculations 2
Assessment of BLYP-D3 Based on Literature 2
Quantum Chemistry Calculations of Reaction Energies 3

S.III. Excess Proton Diffusion in HF and HCl Solutions 4

S.IV. Relation Between the Absorption Coefficient and the Dielectric Spectrum 5

S.V. Obtaining the Dielectric Spectrum from Ab-Initio Molecular Dynamics Simulations 6

S.VI. Extraction of the Difference Spectra from Experimental Data 7

References 8

S.I. COMPUTATIONAL DETAILS

All production simulations were performed in the NVT ensemble at 300 K using CP2K [1]. The BLYP exchange-correlation functional [2, 3] with D3 dispersion corrections [4] was employed. Core electrons were treated with Goedecker-Teter-Hutter (GTH) pseudopotentials [5]. DZVP-MOLOPT-SR-GTH basis sets were used for O and H, and aug-DZVP-GTH for F and Cl [6]. A plane-wave cutoff of 400 Ry was applied. The timestep was 0.5 fs, and temperature control was achieved using a CSVR thermostat [7] with a 100 fs time constant. Simulations contained 256 water molecules for pure water and 224 water molecules for the 7.5 mol/kg acid solutions, with 30 excess protons and 30 anions in the latter. The pure water system was prepared as described in Ref. [8]. Initial HCl structures were generated by classical MD using GROMACS [9]: 200 ps in the NPT ensemble at ambient pressure followed by 800 ps in the NVT ensemble at 300 K, employing the SPC/E water model [10] and Cl^- and H_3O^+ force-field parameters from Ref. [11]. For HF, anions were replaced and configurations rescaled according to experimental density ratios [12, 13]. The first 14 ps (HF) and 9 ps (HCl) were discarded for equilibration. The amount of Zundel- and Eigen structures are found to be in chemical equilibrium after this time. Box lengths were 19.73 Å (pure water), 20.23 Å (7.5 mol/kg HCl), and 19.63 Å (7.5 mol/kg HF). Trajectory lengths were 201 ps (water), 91 ps (HCl), and 128 ps (HF). Water and HCl trajectories were previously analyzed [8, 14] and are reused here for comparison with HF. To simulate a dilute HF solution, a single HF molecule in water was simulated in a 9.855 Å cubic box containing 31 water molecules. Therefore, a stable configuration of 32 water molecules at experimental density (1.0 g/cm^3) was generated via a classical 20 ps NVT simulation as described above (but with a reduced Lennard-Jones cutoff of 4.5 Å), and then equilibrated for 10 ps with AIMD after replacing one water molecule by HF. The remaining 39 ps were used for analysis.

The IR spectra were calculated from the Fourier transformation of the polarization autocorrelation function using Wannier center localization which is briefly described in Ref. [14] and detailed in Sections S.IV and S.V.
Bound protons are defined as the two protons closest to each oxygen atom in the system, the remaining protons are identified as excess protons. Zundel configurations ($H_5O_2^+$, H_3OF, and H_3OCl) are identified based on the two closest heavy atoms (O, F, or Cl) to an excess proton. A similar, but slightly more complex definition of excess protons was used in Ref. [14], which was needed to obtain undisrupted trajectories of excess protons for the spectral analysis of the barrier-crossing dynamics. Eigen configurations are defined by a central H_3O^+. The H_3O^+ oxygen is taken as the oxygen atom closest to the excess proton. Proton sharing anions are defined as the hydrogen bonded neighbors to the H_3O^+ oxygen. Hydrogen bonds are defined by the geometric definition of Ref. [15]. To enforce mass and charge conservation, each anion (F^-, Cl^-) is assigned to its nearest H_3O^+, avoiding double counting. Consequently, H_2O molecules in the Eigen-picture (Fig. 1(b) of the main text) should be understood as generic hydrogen-bond acceptors, which are mostly water but can also be an anion bound to a different H_3O^+. Counting hydrogen-bond partners of each hydronium ion yields the distributions: HF ($H_9O_4^+$ 17%, H_7O_3F 44%, $H_5O_2F_2^-$ 28%) and HCl ($H_9O_4^+$ 64%, H_7O_3Cl 26%, $H_5O_2Cl_2^-$ 3%), which differ from those reported in the main text, since only unique anion assignments to H_3O^+ ions are used there. Since Braddy et al. [16] report only the molalities of H^+ and F^-, the molalities of HF and HF_2^- in the experimental data are obtained from charge conservation, $m_{H^+} = m_{HF_2^-} + m_{F^-}$, and fluorine conservation, $m^0_{HF} = m_{HF} + m_{F^-} + 2 \times m_{HF_2^-}$, where m_α denotes the molality of species α and m^0_{HF} the initial HF molality. This treatment effectively reduces the analysis to the building blocks H^+, HF, F^-, and HF_2^- and does not consider $H_2F_3^-$ formation, which was considered in the analysis of Ref. [16]. This is consistent with our simulation analysis, where we do not investigate aggregation of different Eigen-like structures. The shortest path between the excess proton and the conjugate base is determined by the breadth-first-search algorithm, using the implementation in the Python package NetworkX [17]. We neglect nuclear quantum effects and therefore compute the free-energy landscapes from classical probability distributions. Although nuclear quantum effects can modify these distributions [18–20], they are expected to affect all systems similarly and thus are not expected to alter the presented comparison. Their influence on IR spectra is also small, since classical and quantum harmonic oscillators share the same position response function [21, 22], which explains why no quantum correction factor [23] needs to be applied to the IR spectra in this work, as described in the Supplemental Information of Ref. [24].

S.II. DISCUSSION OF THE EMPLOYED LEVEL OF THEORY AND QUANTUM CHEMISTRY CALCULATIONS

Assessment of BLYP-D3 Based on Literature

The here employed BLYP-D3 level of theory provides a practical compromise between accuracy and computational cost. In a broad benchmark of density functionals for water properties [25], it achieved an overall score of 66/100, outperforming methods such as BLYP (42/100), PBE (56/100), PBE-TS (56/100) [26, 27] and revPBE-DRSLL (49/100) [28, 29], although optPBE-DRSLL and optB88-DRSLL performed better (74/100) [28, 30]. BLYP-D3 has also been shown to accurately reproduce spectral properties of pure water [14] and HCl solutions at various concentrations [8] and is demonstrated to give excellent agreement with the lattice energy of ice polymorphs [31]. Nevertheless, BLYP-D3 has known limitations. It substantially underestimates

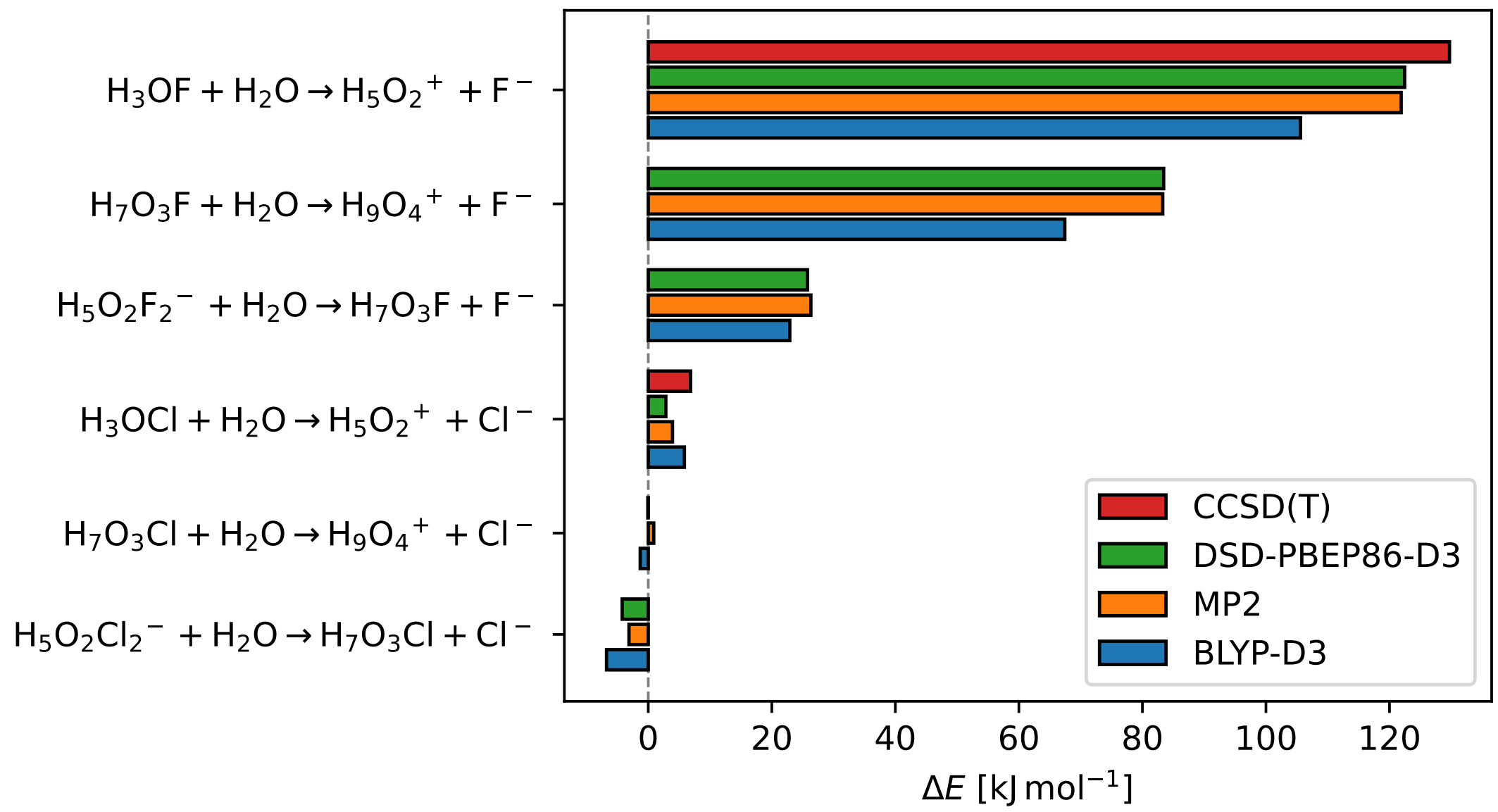

FIG. S1. Reaction energies ΔE of the proton-transfer reactions in the Zundel- and Eigen-picture for different levels of theory, based on quantum chemistry calculations of the energy-optimized structures sketched in Fig. 1(b) of the main text. The reaction equations are shown on the y-axis. The energies are computed using CCSD(T) (red), MP2 (orange), DSD-PBEP86-D3 (green) and BLYP-D3 (blue). Note, that the reaction energies are not directly comparable to experimental values since the solvent is only taken into account in a rather approximative manner and only the minimum-energy geometry of each structure is considered.

the experimental water self-diffusion coefficient $D = 0.23\,\text{Å}^2/\text{ps}$ [32], yielding $D = 0.07 \pm 0.01\,\text{Å}^2/\text{ps}$ in the dataset analyzed here, consistent with previous studies [33]. It also underestimates the proton-transfer barrier in isolated Zundel cations relative to CCSD(T) [34]. Both drawbacks can be corrected using an additional atomic pair potential (EDS-BLYP-D3) [33, 34], which is not parameterized for oxygen-fluoride and hydrogen-fluoride interactions and was therefore not used here. In summary, the literature reports different assessments for the ability of BLYP-D3 to accurately describe aqueous systems. In the following section we report additional quantum-chemical calculations of reaction energies in the Zundel- and Eigen-picture to assess the applicability of BLYP-D3 for the systems studied here.

Quantum Chemistry Calculations of Reaction Energies

Here, we present reaction energies obtained from quantum-chemical calculations involving the Eigen- and Zundel-like structures depicted in Fig. 1(b) of the main text. We note that the reaction energies obtained from these calculations are not directly comparable to experimental values for two reasons: Firstly, the solvent environment is treated only approximately using the conductor-like polarizable continuum model (C-PCM) [35] and, secondly, only the minimum-energy geometry of each structure is considered. Nevertheless, these calculations provide a quantitative assessment of the accuracy of the BLYP-D3 level of theory and allow us to draw qualitative conclusions about the relative stability of the different Zundel- and Eigen-like structures observed in the bulk AIMD simulations. We used ORCA 6.1.0 [36, 37] to perform geometry optimizations of the water molecule H_2O and of the Zundel cation $H_5O_2^+$, the Eigen cation $H_9O_4^+$, as well as H_3OX, H_7O_3X, and $H_5O_2X_2^-$, with $X \in \mathrm{F}, \mathrm{Cl}$. For H and O, we employed the cc-pVTZ basis set [38], whereas for F and Cl we used aug-cc-pVTZ [38–40]. We note that the comparatively small basis set used in the bulk AIMD simulations constitutes an additional source of error that is not considered in the following analysis. We repeated these calculations at four different levels of theory using tight convergence thresholds for the self-consistent field energies and gradients. The considered levels of theory are BLYP-D3, consisting of BLYP DFT with Grimme's D3 dispersion correction [41, 42] and Becke–Johnson damping [43, 44], MP2, based on second-order Møller–Plesset perturbation theory [45] using the resolution-of-the-identity (RI) approximation, DSD-PBEP86-D3, employing the double-hybrid DSD-PBEP86 functional with D3 dispersion and Becke–Johnson damping [46–48] and RI for the MP2 correlation contribution, and CCSD(T), including coupled-cluster singles, doubles, and perturbative triples [49] with numerical gradients. CCSD(T) serves as the reference method. However, because of its high computational cost, for the larger systems MP2 and DSD-PBEP86-D3 are used as more affordable alternatives.

We define $\Delta E = E_\mathrm{B} - E_\mathrm{A}$ for the reaction $\mathrm{A} \rightarrow \mathrm{B}$, where E_A and E_B are the internal energies of the reactant and product states A and B, respectively. The proton-transfer energy is computed by replacing the conjugate base in each proton-bearing species

with a water molecule. The corresponding reaction equations are given on the y-axis of Fig. S1. We consider the Zundel-like and Eigen-like structures depicted in Fig. 1(b) of the main text and compare the resulting proton-transfer energies obtained at the different levels of theory in Fig. S1. Overall, BLYP-D3 reproduces the energy differences obtained at the other levels of theory reasonably well, as shown by the agreement between the BLYP-D3 results (blue bars) and those obtained with CCSD(T) (red), MP2 (orange), and DSD-PBEP86-D3 (green). However, BLYP-D3 underestimates the binding energy of an excess proton to fluoride, which suggests that the predicted equilibrium between the Zundel- and Eigen-like structures shown in Fig. 1(b) of the main text likely underestimates the fraction of excess protons coordinated to fluoride. Coordinates of the optimized structures are provided in Tables S1-S31. In the following, the individual proton-transfer reactions are discussed briefly. The first reaction considered is proton transfer in the Zundel picture

$$H_3OX + H_2O \rightarrow H_5O_2^+ + X^- \,. \tag{S1}$$

For $X^- = F^-$, the proton-transfer energy is strongly positive at all levels of theory, whereas for $X^- = Cl^-$ it is substantially smaller, which is consistent with the thermodynamic expectation that HF is a much weaker acid than HCl. The same observation can be made for the proton-transfer reaction in the Eigen picture, which differs from the Zundel picture by the presence of two additional water molecules in the proton-bearing species

$$H_7O_3X + H_2O \rightarrow H_9O_4^+ + X^- \,. \tag{S2}$$

Note, that the reaction in the Eigen-picture in Eq. (S2) and in the Zundel-picture in Eq. (S1) both describe the same reaction, but only differ in the number of water molecules explicitly included in the quantum-chemical calculations. Interestingly, the reaction energy is smaller for both anions in the Eigen picture than in the Zundel picture. Since the dielectric contribution of the aqueous solvent is already accounted for through C-PCM, this additional stabilization must originate from explicit chemical interactions with the surrounding water molecules. This, in turn, suggests that the Eigen picture provides a more appropriate description of the equilibrium structure of the hydrated excess proton, in agreement with Ref. [34] and our interpretation of the bulk AIMD simulations. Finally, we consider the ligand exchange energy for the Eigen-like difluoride $H_5O_2F_2{}^-$ and dichloride $H_5O_2Cl_2{}^-$ anions, in which one anion is replaced by a water molecule to form the corresponding Eigen-like structure containing a single anion

$$H_5O_2X_2^- + H_2O \rightarrow H_7O_3X + X^- \,. \tag{S3}$$

We find that an excess proton is strongly stabilized by an additional fluoride ion, whereas the stabilization provided by an additional chloride ion is substantially weaker. This result indicates that a single excess proton can be stabilized by multiple fluoride ions, consistent with our bulk AIMD findings presented in the main text.

S.III. EXCESS PROTON DIFFUSION IN HF AND HCL SOLUTIONS

Here, we describe the calculation of the diffusion constants of excess protons in the concentrated HF and HCl solutions from the AIMD simulations and interpet the results. The diffusion coefficient D is obtained from the mean-square displacement (MSD) of the excess protons according to

$$D = \lim_{t\to\infty} \frac{1}{6t} \langle |\boldsymbol{r}(t) - \boldsymbol{r}(0)|^2 \rangle \,, \tag{S4}$$

[50], where $\boldsymbol{r}(t)$ is the position and $\langle\cdot\rangle$ denotes an ensemble average. We define excess protons as described in Section S.I. To determine the position of an excess proton, we assign it to the closest heavy atom (O, F, or Cl). We then track the motion of the excess proton by following the heavy atom to which it is associated, as long as no proton transfer occurs. To identify proton transfer, we monitor all protons covalently bound to this heavy atom. When one of these protons is transferred to another heavy atom, the receiving heavy atom becomes the new reference site, and the trajectory of the excess proton is continued from there. In rare cases, simultaneous proton-transfer events occur, such that the receiving heavy atom immediately transfers a proton to a third heavy atom and therefore no longer carries the excess charge. In these cases, we follow the subsequent receiving heavy atom and continue the excess-proton trajectory from that site. This implementation is similar to the one in Ref. [14]. Our results for HCl and H_2O are consistent with those in Ref. [14], but HF was not considered in that work. As in Ref. [14], we fit the mean-square displacement (MSD) to a linear function for $t > 20\,\text{ps}$ to determine the diffusion coefficient. These fits alongside the time dependend MSD are shown in Fig. S2a. We find that the diffusion coefficient of excess protons in the HF solution is $D_{\text{HF}}^{\text{H}^+} = 0.09\,\text{Å}^2/\text{ps}$, which is way smaller than in the HCl solution $D_{\text{HCl}}^{\text{H}^+} = 0.26\,\text{Å}^2/\text{ps}$. For reference, we obtain a water diffusion coefficient of $D_{\text{H}_2\text{O}}^{\text{H}_2\text{O}} = 0.07\,\text{Å}^2/\text{ps}$, substantially smaller than the experimental value as already mentioned in Section S.II. Calio

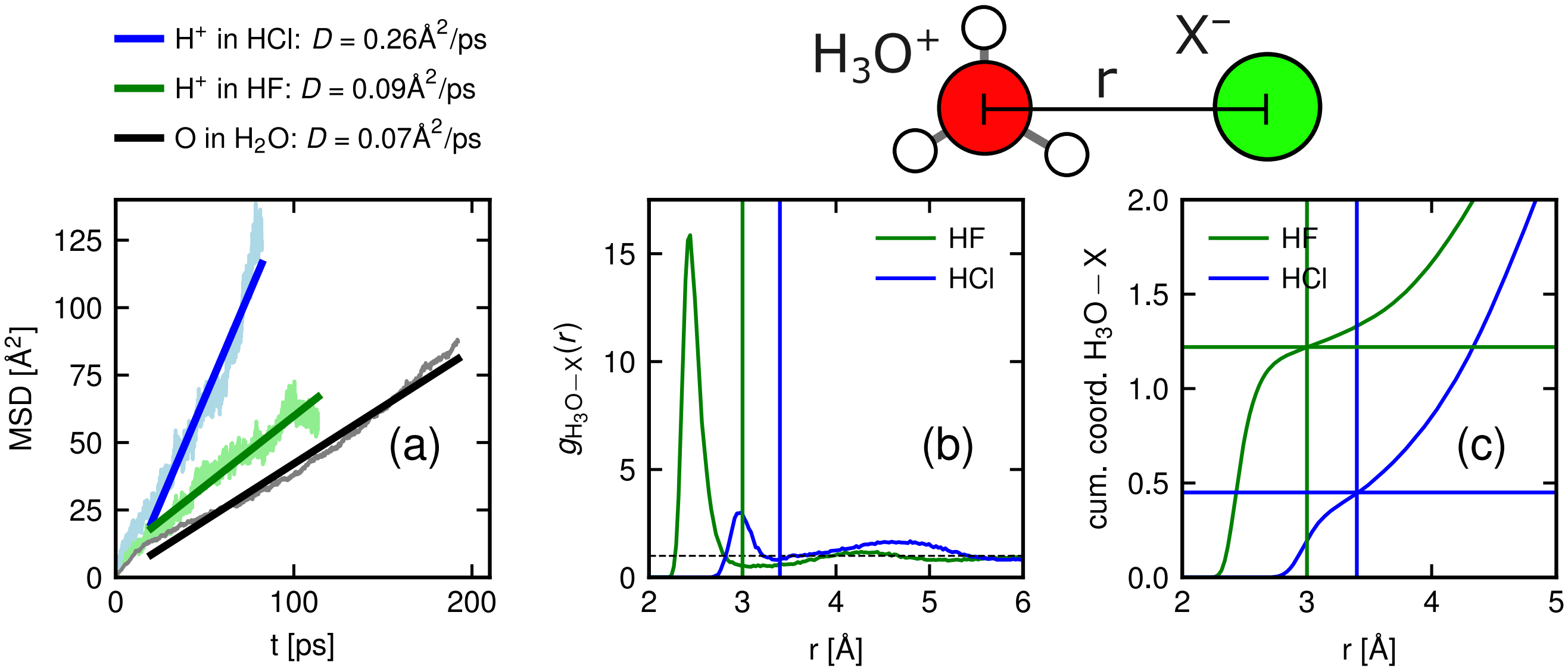

FIG. S2. (a): Mean-square displacement (MSD) of excess protons in the 7.5 mol/kg HF (green) and HCl (blue) solutions as a function of time, compared with the MSD of water molecules in pure water (black). The dark lines show linear fits to the MSD data for $t > 20$, ps, from which the corresponding diffusion coefficients are determined. The lighter lines show the calculated MSD trajectories. (b): Radial distribution function between hydronium oxygen atoms and the conjugate base anions F^- (green) and Cl^- (blue) in the HF and HCl solutions, respectively. (c): Cumulative coordination number of the conjugate base anions around hydronium oxygen atoms in the HF and HCl solutions, obtained by integrating the radial distribution functions shown in panel (b). The vertical lines in panels (b) and (c) indicate the first coordination shell and the horizontal lines in panel (c) indicate the corresponding coordination numbers.

et al. used temperature-dependent radial distribution functions to elucidate the molecular origins of proton-transfer barriers in HCl solutions [51]. Direct comparison is not possible because our simulations are available only at a single temperature, but the solvation structure can still be characterized through the radial distribution function between hydronium oxygen atoms and the conjugate-base anions, shown in Fig. S2(b). Here, ”hydronium oxygen” denotes the oxygen atom closest to the excess proton, even when a conjugate-base anion lies closer to the proton, as frequently occurs in HF solutions. Figure S2(b) reveals pronounced differences in the hydronium solvation structure between HF and HCl solutions. The first peak of the H_3O^+-F^- radial distribution function is located at 2.4 Å, which is significantly smaller than the corresponding H_3O^+-Cl^- distance at 3 Å. The radial distribution of the hydronium oxygen and Cl^- agrees well with the one reported by Calio et al. [51]. Further, the peak height of the H_3O^+-F^- radial distribution function is substantially larger than that of the H_3O^+-Cl^- radial distribution function. Together, these observations demonstrate that excess protons are strongly coordinated to fluoride ions, whereas chloride ions are only weakly coordinated to excess protons, in agreement with thermodynamic expectation and the quantum-chemical calculations of the reaction energies in Section S.II. By integrating the radial distribution functions, we obtain the cumulative coordination number of the conjugate base anions around hydronium oxygen atoms, which is shown in Fig. S2(c). We find that within the first solvation shell of hydronium oxygen atoms, there are on average 1.22 fluoride ions and only 0.45 chloride ions. Together, these results demonstrate that excess protons are strongly coordinated to fluoride ions, which explains why the mobility of excess protons in HF solutions is significantly lower and demonstrates that our AIMD simulations capture the expected thermodynamic behavior of weak and strong acid solutions.

S.IV. RELATION BETWEEN THE ABSORPTION COEFFICIENT AND THE DIELECTRIC SPECTRUM

We consider the one-dimensional inhomogeneous wave equation in vacuum

$$\left(\frac{\partial^2}{\partial z^2} + \frac{\omega^2}{c^2}\right) E(z,t) = \mu_0 \dot{J}(z,t), \tag{S5}$$

with a current density $J(z,t)$ induced by a sinusoidal electric field given by $E(z,t) = \mathcal{E}(z)e^{-i\omega t} + c.c.$. Here c is the speed of light in vacuum, μ_0 the vacuum permeability [52], $c.c.$ is the complex conjugate and $\mathcal{E}(z)$ the z-dependent amplitude of the electric

field. If the electric field is sufficiently weak, the current density is related to the electric field by the constitutive relation

$$J(z,t) = \varepsilon_0 \tilde{\sigma}_{\mathrm{P}}(\omega)\mathcal{E}(z)e^{-i\omega t} + c.c.\,, \tag{S6}$$

where $\tilde{\sigma}_{\mathrm{P}}(\omega)$ is a complex frequency-dependent conductivity and ε_0 is the vacuum permittivity. We associate all charges in our system to the polarization density $P(z,t)$ which obeys

$$\dot{P}(z,t) = J(z,t)\,. \tag{S7}$$

We note that the conductivity $\tilde{\sigma}_{\mathrm{P}}(\omega)$ is the polarization conductivity and is thus different from the usual conductivity, which only accounts for transport of free charges in response to an electric field [53]. Introducing the complex dielectric function as

$$\omega\tilde{\varepsilon}(\omega) = \omega + i\tilde{\sigma}_{\mathrm{P}}(\omega)\,, \tag{S8}$$

we obtain from Eqs (S6) and (S7)

$$J(z,t) = \dot{P}(z,t) = -i\omega\varepsilon_0\left(\tilde{\varepsilon}(\omega) - 1\right)\mathcal{E}(z)e^{-i\omega t} + c.c.\,. \tag{S9}$$

With this, we eliminate the current density in Eq. (S5) and obtain the homogeneous wave equation in matter

$$\left(\frac{\partial^2}{\partial z^2} + \tilde{k}(\omega)^2\right)E(z,t) = 0\,, \tag{S10}$$

where we introduced the complex wave vector $\tilde{k}(\omega) = \frac{\omega}{c}\sqrt{\tilde{\varepsilon}(\omega)}$ and used the relation $c^2 = 1/(\varepsilon_0\mu_0)$ [52]. The solution for the electric field is given by

$$E(z,t) = \mathcal{E}_0 e^{-i\left(\omega t \pm \tilde{k}(\omega)z\right)} + c.c.\,, \tag{S11}$$

where the sign in the exponent determines the direction of propagation and $\mathcal{E}_0$ is the z-independent amplitude. The absorption coefficient $\tilde{\alpha}(\omega)$ is defined as the inverse length at which the intensity of the electric field decreases by a factor $1/e$ and is thus given by

$$\tilde{\alpha}(\omega) = 2\tilde{k}(\omega)''\,, \tag{S12}$$

where $\tilde{k}(\omega)''$ is the imaginary part of the complex wave vector. The factor 2 appears because the intensity is proportional to the square of the electric field.

S.V. OBTAINING THE DIELECTRIC SPECTRUM FROM AB-INITIO MOLECULAR DYNAMICS SIMULATIONS

We consider an overall charge-neutral system of N atoms with charges q_i, and electric dipole moments $\mu_i(t)$, occupying a volume V under periodic boundary conditions. The current density is given by [53]

$$J(t) = \frac{1}{V}\sum_{i=1}^{N}\left(q_i v_i(t) + \dot{\mu}_i(t)\right)\,. \tag{S13}$$

In our case the oxygen, hydrogen, fluoride and chloride atoms carry the charges $-2e$, e, $-e$ and $-e$, respectively. In order to account for electronic polarization we conduct Wannier center localization. Each Wannier center has the charge $-2e$. Four Wannier centers are unambiguously assigned to the closest heavy atom (O,F,Cl) and zero to hydrogen in agreement with the oktet rule. Therefore, the electric dipole moment of atom i is given by

$$\mu_i(t) = -2e\sum_{j=1}^{N_i^{\mathrm{WC}}}\left(r_{ij}^{\mathrm{WC}}(t) - r_i(t)\right)\,. \tag{S14}$$

Here, $r_{ij}^{\mathrm{WC}}(t)$ is the position of the j-th Wannier center assigned to atom i, $r_i(t)$ is the position of atom i and N_i^{WC} is the number of Wannier centers assigned to atom i. The distance vector $r_{ij}^{\mathrm{WC}}(t) - r_i(t)$ is defined as the shortest vector between the Wannier center and the atom, accounting for periodic boundary conditions. We define the polarization density as

$$P(t) = \frac{1}{V}\sum_{i=1}^{N}\left(q_i r_i(t) + \mu_i(t)\right)\,, \tag{S15}$$

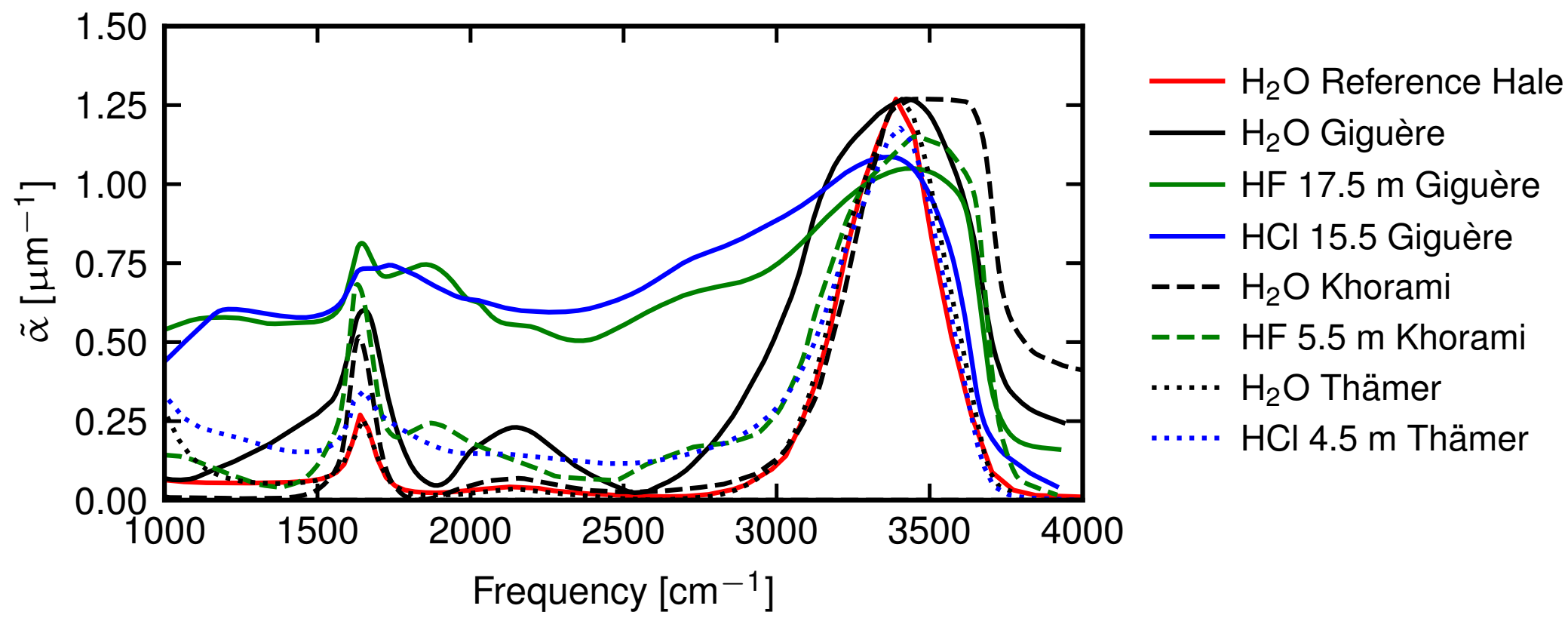

FIG. S3. Comparison of experimental absorption spectra from the literature [58–60]. Pure water spectra are shown in black, HF solutions in green, and HCl solutions in blue. For reference, the well-established literature absorption coefficient of pure water [61] is shown in red and agrees best with the pure-water spectrum from Thämer *et al.* [60]. While the experimental pure-water spectra differ considerably between the datasets, the spectral signatures of the excess protons are sufficiently pronounced and robust to enable a meaningful comparison across the different measurements.

where $r_i(t)$ denotes the position of atom i. In contrast to $J(t)$ defined in Eq. (S13), $P(t)$ does depend on the choice of the atomic origins, which we choose to be the nuclei positions. For consistency with the definition of the current density in Eq. (S13), the time derivative of the position must satisfy $\dot{r}_i(t) = v_i(t)$. Under periodic boundary conditions, this requires the use of unwrapped atomic positions, i.e., positions that are made continuous in time by adding or subtracting the box length whenever an atom crosses a periodic boundary. We pick the initial atomic position $r_i(0)$ such that all atoms are inside the simulation box at time $t = 0$, but other choices are possible as long as $r_i(t)$ is continuous in time. In bulk systems, the external field in the perturbation Hamiltonian can be identified as the electric field [54] and we can use the fluctuation dissipation theorem [21, 55] to relate the imaginary part of the dielectric function to the Fourier transformed trajectory of the polarization density of length τ as

$$\omega\tilde{\varepsilon}''(\omega) = \omega^2 \frac{V}{2\varepsilon_0 k_B T \tau} |\tilde{P}(\omega)|^2 . \tag{S16}$$

If the system is isotropic, Eq. (S16) can be averaged over the three dimensions. Using Eq. (S7) we could also express the imaginary part of the dielectric function in terms of the current density as

$$\omega\tilde{\varepsilon}''(\omega) = \frac{V}{2\varepsilon_0 k_B T \tau} |\tilde{J}(\omega)|^2 . \tag{S17}$$

Since the Wannier center localization procedure is numerically expensive, it is not numerically feasable to compute the atomic dipole moments at every time step. Therefore, $J(t)$ can only be computed from a highly discretized time series of $P(t)$, which leads to significant time discretization errors in Eq. (S17). This problem can be circumvented by using Eq. (S16) instead of Eq. (S17) to compute the imaginary part of the dielectric function, where time discretization is not an issue as long as the Nyquist–Shannon sampling criterion is fulfilled, i.e., as long as the sampling frequency exceeds twice the highest frequency present in the signal [56]. In order to compute the absorption coefficient defined in Eq. (S12) we also need the real part of the dielectric function, which is obtained by numerical Kramers-Kronig transformation as described elsewhere [24] and where we add the high-frequency contribution $\varepsilon^{\mathrm{VIS}} = 1.78$ [57] to the real part of the dielectric function to account for electronic polarizability.

S.VI. EXTRACTION OF THE DIFFERENCE SPECTRA FROM EXPERIMENTAL DATA

All experimental spectra we compare our simulated spectra with are reported without units in the original works [58–60]. In the dataset by Thämer et al. [60], the zero baseline is clearly identifiable for the pure-water and the 4.5 m HCl spectra, therefore both spectra can be straightforwardly brought onto an absolute scale by multiplication with a factor that is chosen such that the maximum of the pure-water absorption spectrum for the OH-stretch peak matches the spectrum from Hale et al. [61] for which units are given. As evident from Fig. S3, the pure water spectrum from Thämer et al. [60], (black dotted line) agrees quantitatively with the spectrum from Ref. [61] (red solid line), and therefore we consider it to be the most reliable dataset available. Except for the pure-water spectrum reported by Giguère and Turrell [58], for which we assume the boundary

of the plotted spectral window to correspond to zero intensity, the spectra in Refs. [58, 59] are vertically shifted by unknown offsets, and neither units nor zero lines are specified in the original works. Because pure water does not exhibit strong broad continuum bands (red line in Fig. S3), the pure-water spectrum of Khorami et al. [59] (black dashed line) is shifted such that its minimal intensity becomes equal to zero. In contrast, the broad continuum absorption of the acid spectra from Khorami et al. and Giguère and Turrell prevents the identification of a reliable minimum that could serve as a zero-intensity reference. Alignment at 4000 cm^{-1} is likewise not justified because of the pronounced background, which has been attributed to reflectivity and interference effects in similar experimental setups [62]. This background was also explicitly noted by Giguère and Turrell [63] and is evident from the substantial nonzero intensities of the pure-water spectra near 4000 cm^{-1} reported by Khorami et al. (black dashed line) and Giguère and Turrell (black solid line). In the absence of a clearly identifiable absolute zero line for these acid-solution spectra, our procedure is to align them based on the high-quality and zero-aligned 4.5 m HCl spectrum of Thämer et al. [60], which is shown in Fig. S3 as a dotted blue line. In particular, we observe that HCl and HF solutions at comparable concentrations exhibit very similar line shapes. This holds for the 4.5 m HCl spectrum of Thämer et al. (dotted blue line) and the 5.5 m HF spectrum of Khorami et al. (dashed green line), and is also apparent for the high-concentration HF and HCl spectra of Giguère and Turrell (solid green and blue lines). We therefore align the 5.5 m HF spectrum of Khorami et al. with the 4.5 m HCl spectrum of Thämer et al., while the high-concentration HF and HCl spectra of Giguère and Turrell are aligned relative to each other. The remaining absolute position of the zero line is chosen such that the overall continuum intensity scales approximately with acid concentration, using the Thämer HCl data as the reference. The constant vertical offset is the only parameter required, since in the datasets of Khorami et al. and Giguère and Turrell all spectra are shown in the same figure and therefore share the same intensity scale. The absolute intensity scale of each dataset is determined unambiguously by multiplication with a single factor such that the maximum of the corresponding pure-water spectrum matches the maximum of the Hale reference spectrum. In fact, the uncertainty produced by our rescaling procedure could only be resolved by new experimental spectra that report a clearly identifiable absolute zero. Given the available data, our alignment procedure described above provides the most straightforward way of fixing this ambiguity. A residual constant offset between the HF and HCl spectra would amount to a frequency-independent contribution to the absorption, which would be difficult to justify physically. Also, our shifting of the zero line cannot be the cause of the observed agreement of the line shapes of HF and HCl. In particular, a constant vertical shift does not modify the relative intensities of the broad bands between 1000–1500 cm^{-1}, 1800–3000 cm^{-1} and water OH-stretching band between 3000–4000 cm^{-1}, which are especially evident in the high-concentration HF and HCl spectra of Giguère and Turrell (solid green and blue lines in Fig. S3), but can also be seen in the 5.5 m HF spectrum of Khorami et al. (dashed green line) and the 4.5 m HCl spectrum of Thämer et al. (dotted blue line). We compute the experimental difference spectra by subtracting the corresponding pure-water spectrum from each acid-solution spectrum according to Eq. (3) of the main text, using the experimental solution densities reported in Refs. [57, 64] for the normalization. Overall, although the quality of the absolute absorption spectra varies between the available experimental datasets, the pronounced spectral signatures of the acids allow for robust comparison between our simulations and the experimental data. Given the larger experimental uncertainties in the older datasets, future measurements of hydrohalic acids at the same level as those from Thämer et al. [60] would be desirable to enable a direct quantitative analysis of the experimental spectral signatures of the excess proton in weak and strong acid solutions.

---

[1] T. D. Kühne, M. Iannuzzi, M. Del Ben, V. V. Rybkin, P. Seewald, F. Stein, T. Laino, R. Z. Khaliullin, O. Schütt, F. Schiffmann, D. Golze, J. Wilhelm, S. Chulkov, M. H. Bani-Hashemian, V. Weber, U. Borštnik, M. Taillefumier, A. S. Jakobovits, A. Lazzaro, H. Pabst, T. Müller, R. Schade, M. Guidon, S. Andermatt, N. Holmberg, G. K. Schenter, A. Hehn, A. Bussy, F. Belleflamme, G. Tabacchi, A. Glöß, M. Lass, I. Bethune, C. J. Mundy, C. Plessl, M. Watkins, J. VandeVondele, M. Krack, and J. Hutter, CP2K: An electronic structure and molecular dynamics software package - Quickstep: Efficient and accurate electronic structure calculations, The Journal of Chemical Physics **152**, 194103 (2020).

[2] A. D. Becke, Density-functional exchange-energy approximation with correct asymptotic behavior, Physical Review A **38**, 3098 (1988).

[3] C. Lee, W. Yang, and R. G. Parr, Development of the Colle-Salvetti correlation-energy formula into a functional of the electron density, Physical Review B **37**, 785 (1988).

[4] S. Grimme, J. Antony, S. Ehrlich, and H. Krieg, A consistent and accurate ab initio parametrization of density functional dispersion correction (DFT-D) for the 94 elements H-Pu, The Journal of Chemical Physics **132**, 154104 (2010).

[5] S. Goedecker, M. Teter, and J. Hutter, Separable dual-space Gaussian pseudopotentials, Physical Review B **54**, 1703 (1996).

[6] J. VandeVondele and J. Hutter, Gaussian basis sets for accurate calculations on molecular systems in gas and condensed phases, The Journal of Chemical Physics **127**, 114105 (2007).

[7] G. Bussi, D. Donadio, and M. Parrinello, Canonical sampling through velocity rescaling, The Journal of Chemical Physics **126**, 014101 (2007).

[8] F. N. Brünig, O. Geburtig, A. von Canal, J. Kappler, and R. R. Netz, Time-Dependent Friction Effects on Vibrational Infrared Frequencies and Line Shapes of Liquid Water, The Journal of Physical Chemistry B **126**, 1579 (2022).

[9] M. J. Abraham, T. Murtola, R. Schulz, S. Páll, J. C. Smith, B. Hess, and E. Lindahl, GROMACS: High performance molecular simulations through multi-level parallelism from laptops to supercomputers, SoftwareX **1–2**, 19 (2015).

[10] H. J. C. Berendsen, J. R. Grigera, and T. P. Straatsma, The missing term in effective pair potentials, The Journal of Physical Chemistry **91**, 6269 (1987).

[11] D. J. Bonthuis, S. I. Mamatkulov, and R. R. Netz, Optimization of classical nonpolarizable force fields for OH- and H3O+, The Journal of Chemical Physics **144**, 104503 (2016).

[12] S. L. Clegg and A. S. Wexler, Densities and apparent molar volumes of atmospherically important electrolyte solutions. 1. The solutes H2SO4, HNO3, HCl, Na2SO4, NaNO3, NaCl, (NH4)2SO4, NH4NO3, and NH4Cl from 0 to 50 °C, including extrapolations to very low temperature and to the pure liquid state, and NaHSO4, NaOH, and NH3 at 25 °C, The Journal of Physical Chemistry. A **115**, 3393 (2011).

[13] Acid & Base Normality and Molarity Calculator, https://www.sigmaaldrich.com/DE/de/support/calculators-and-apps/molarity-calculator.

[14] F. N. Brünig, M. Rammler, E. M. Adams, M. Havenith, and R. R. Netz, Spectral signatures of excess-proton waiting and transfer-path dynamics in aqueous hydrochloric acid solutions, Nature Communications **13**, 4210 (2022).

[15] A. Ozkanlar, T. Zhou, and A. E. Clark, Towards a unified description of the hydrogen bond network of liquid water: A dynamics based approach, The Journal of Chemical Physics **141**, 214107 (2014).

[16] R. Braddy, P. T. McTigue, and B. Verity, Equilibria in moderately concentrated aqueous hydrogen fluoride solutions, Journal of Fluorine Chemistry **66**, 63 (1994).

[17] A. Hagberg, P. J. Swart, and D. A. Schult, *Exploring Network Structure, Dynamics, and Function Using NetworkX*, Tech. Rep. LA-UR-08-05495; LA-UR-08-5495 (Los Alamos National Laboratory (LANL), 2008).

[18] U. W. Schmitt and G. A. Voth, The computer simulation of proton transport in water, The Journal of Chemical Physics **111**, 9361 (1999).

[19] D. Marx, Proton Transfer 200 Years after von Grotthuss: Insights from Ab Initio Simulations, ChemPhysChem **7**, 1848 (2006).

[20] A. O. Atsango, T. Morawietz, O. Marsalek, and T. E. Markland, Developing machine-learned potentials to simultaneously capture the dynamics of excess protons and hydroxide ions in classical and path integral simulations, The Journal of Chemical Physics **159**, 074101 (2023).

[21] R. Kubo, The fluctuation-dissipation theorem, Reports on Progress in Physics **29**, 255 (1966).

[22] A. O. Caldeira and A. J. Leggett, Quantum tunnelling in a dissipative system, Annals of Physics **149**, 374 (1983).

[23] R. Ramírez, T. López-Ciudad, P. Kumar P, and D. Marx, Quantum corrections to classical time-correlation functions: Hydrogen bonding and anharmonic floppy modes, The Journal of Chemical Physics **121**, 3973 (2004).

[24] L. Lehmann, M. R. Becker, L. Tepper, A. P. Fellows, Á. Diaz Duque, M. Thämer, M. Wolf, and R. R. Netz, Multipolar electric and magnetic contributions to sum-frequency generation spectra reveal biaxial interfacial water structure, Nature Communications **17**, 4333 (2026).

[25] M. J. Gillan, D. Alfè, and A. Michaelides, Perspective: How good is DFT for water?, The Journal of Chemical Physics **144**, 130901 (2016).

[26] J. P. Perdew, K. Burke, and M. Ernzerhof, Generalized Gradient Approximation Made Simple, Physical Review Letters **77**, 3865 (1996).

[27] A. Tkatchenko and M. Scheffler, Accurate Molecular Van Der Waals Interactions from Ground-State Electron Density and Free-Atom Reference Data, Physical Review Letters **102**, 073005 (2009).

[28] M. Dion, H. Rydberg, E. Schröder, D. C. Langreth, and B. I. Lundqvist, Van der Waals Density Functional for General Geometries, Physical Review Letters **92**, 246401 (2004).

[29] Y. Zhang and W. Yang, Comment on "Generalized Gradient Approximation Made Simple", Physical Review Letters **80**, 890 (1998).

[30] J. Klimeš, D. R. Bowler, and A. Michaelides, Chemical accuracy for the van der Waals density functional, Journal of Physics: Condensed Matter **22**, 022201 (2009).

[31] J. G. Brandenburg, T. Maas, and S. Grimme, Benchmarking DFT and semiempirical methods on structures and lattice energies for ten ice polymorphs, The Journal of Chemical Physics **142**, 124104 (2015).

[32] K. R. Harris and L. A. Woolf, Pressure and temperature dependence of the self diffusion coefficient of water and oxygen-18 water, Journal of the Chemical Society, Faraday Transactions 1: Physical Chemistry in Condensed Phases **76**, 377 (1980).

[33] P. B. Calio, G. M. Hocky, and G. A. Voth, Minimal Experimental Bias on the Hydrogen Bond Greatly Improves Ab Initio Molecular Dynamics Simulations of Water, Journal of Chemical Theory and Computation **16**, 5675 (2020).

[34] P. B. Calio, C. Li, and G. A. Voth, Resolving the Structural Debate for the Hydrated Excess Proton in Water, Journal of the American Chemical Society **143**, 18672 (2021).

[35] V. Barone and M. Cossi, Quantum Calculation of Molecular Energies and Energy Gradients in Solution by a Conductor Solvent Model, The Journal of Physical Chemistry A **102**, 1995 (1998).

[36] F. Neese, F. Wennmohs, U. Becker, and C. Riplinger, The ORCA quantum chemistry program package, The Journal of Chemical Physics **152**, 224108 (2020).

[37] F. Neese, Software update: The ORCA program system—Version 5.0, WIREs Computational Molecular Science **12**, e1606 (2022).

[38] T. H. Dunning, Gaussian basis sets for use in correlated molecular calculations. I. The atoms boron through neon and hydrogen, The Journal of Chemical Physics **90**, 1007 (1989).

[39] R. A. Kendall, T. H. Dunning, and R. J. Harrison, Electron affinities of the first-row atoms revisited. Systematic basis sets and wave functions, The Journal of Chemical Physics **96**, 6796 (1992).

[40] D. E. Woon and T. H. Dunning, Gaussian basis sets for use in correlated molecular calculations. III. The atoms aluminum through argon, The Journal of Chemical Physics **98**, 1358 (1993).

[41] S. Grimme, J. Antony, S. Ehrlich, and H. Krieg, A consistent and accurate *ab initio* parametrization of density functional dispersion correction (DFT-D) for the 94 elements H-Pu, The Journal of Chemical Physics **132**, 154104 (2010).

[42] S. Grimme, S. Ehrlich, and L. Goerigk, Effect of the damping function in dispersion corrected density functional theory, Journal of Computational Chemistry **32**, 1456 (2011).

[43] A. D. Becke and E. R. Johnson, A density-functional model of the dispersion interaction, The Journal of Chemical Physics **123**, 154101 (2005).

[44] E. R. Johnson and A. D. Becke, A post-Hartree–Fock model of intermolecular interactions, The Journal of Chemical Physics **123**, 024101 (2005).
[45] Chr. Møller and M. S. Plesset, Note on an Approximation Treatment for Many-Electron Systems, Physical Review **46**, 618 (1934).
[46] S. Kozuch and J. M. L. Martin, DSD-PBEP86: In search of the best double-hybrid DFT with spin-component scaled MP2 and dispersion corrections, Physical Chemistry Chemical Physics **13**, 20104 (2011).
[47] S. Kozuch and J. M. L. Martin, Spin-component-scaled double hybrids: An extensive search for the best fifth-rung functionals blending DFT and perturbation theory, Journal of Computational Chemistry **34**, 2327 (2013).
[48] L. Goerigk, A. Hansen, C. Bauer, S. Ehrlich, A. Najibi, and S. Grimme, A look at the density functional theory zoo with the advanced GMTKN55 database for general main group thermochemistry, kinetics and noncovalent interactions, Physical Chemistry Chemical Physics **19**, 32184 (2017).
[49] G. D. Purvis and R. J. Bartlett, A full coupled-cluster singles and doubles model: The inclusion of disconnected triples, The Journal of Chemical Physics **76**, 1910 (1982).
[50] A. Einstein, über die von der molekularkinetischen Theorie der Wärme geforderte Bewegung von in ruhenden Flüssigkeiten suspendierten Teilchen, Annalen der Physik **322**, 549 (1905).
[51] P. B. Calio, C. Li, and G. A. Voth, Molecular Origins of the Barriers to Proton Transport in Acidic Aqueous Solutions, The Journal of Physical Chemistry B **124**, 8868 (2020).
[52] M. Born and E. Wolf, *Principles of Optics: Electromagnetic Theory of Propagation, Interference and Diffraction of Light*, 7th ed. (Cambridge University Press, Cambridge, 1999).
[53] J. D. Jackson, *Classical Electrodynamics, International Adaptation* (Wiley, Hoboken, 2021).
[54] H. A. Stern and S. E. Feller, Calculation of the dielectric permittivity profile for a nonuniform system: Application to a lipid bilayer simulation, The Journal of Chemical Physics **118**, 3401 (2003).
[55] S. Carlson, F. N. Brünig, P. Loche, D. J. Bonthuis, and R. R. Netz, Exploring the Absorption Spectrum of Simulated Water from MHz to Infrared, The Journal of Physical Chemistry A **124**, 5599 (2020).
[56] C. Shannon, Communication in the Presence of Noise, Proceedings of the IRE **37**, 10 (1949).
[57] W. M. Haynes, *CRC Handbook of Chemistry and Physics* (Taylor & Francis, Boca Raton, 2015).
[58] P. A. Giguère and S. Turrell, H3O+ ions in aqueous acid solutions. The infrared spectra revisited, Canadian Journal of Chemistry **54**, 3477 (1976).
[59] J. Khorami, R. Beaudoin, and H. Ménard, étude des mélanges eau–HF par spectroscopie infrarouge à transformée de Fourier, Canadian Journal of Chemistry **65**, 817 (1987).
[60] M. Thämer, L. De Marco, K. Ramasesha, A. Mandal, and A. Tokmakoff, Ultrafast 2D IR spectroscopy of the excess proton in liquid water, Science **350**, 78 (2015).
[61] G. M. Hale and M. R. Querry, Optical Constants of Water in the 200-nm to 200-μm Wavelength Region, Applied Optics **12**, 555 (1973).
[62] P. Ayotte, M. Hébert, and P. Marchand, Why is hydrofluoric acid a weak acid?, The Journal of Chemical Physics **123**, 184501 (2005).
[63] P. A. Giguere and S. Turrell, The nature of hydrofluoric acid: A spectroscopic study of the proton-transfer complex $H_3O^+ \cdots F^-$, Journal of the American Chemical Society **102**, 5473 (1980).
[64] D. Green and M. Z. Southard, *Perry's Chemical Engineers' Handbook* (McGraw-Hill Education Ltd, New York, N.Y, 2019).

TABLE S1. Optimized Cartesian coordinates of the BLYP-D3 H_2O structure.

atom	x [Å]	y [Å]	z [Å]
O	0.000 000 000 000 00	−0.000 000 000 048 66	0.011 469 818 875 09
H	0.000 000 000 000 00	0.762 640 884 640 80	−0.592 814 909 457 19
H	0.000 000 000 000 00	−0.762 640 884 592 16	−0.592 814 909 417 89

TABLE S2. Optimized Cartesian coordinates of the BLYP-D3 $H_5O_2^+$ structure.

atom	x [Å]	y [Å]	z [Å]
H	0.198 942 012 818 41	−0.001 224 731 594 11	−0.000 164 544 097 42
O	0.244 863 321 362 40	0.147 882 923 186 63	1.209 558 557 752 96
H	0.191 383 207 762 92	−0.721 376 114 088 92	1.657 617 708 899 04
H	−0.530 707 679 250 22	0.663 241 781 329 77	1.512 993 845 502 87
O	0.240 476 344 493 61	−0.151 246 603 527 96	−1.210 117 182 270 15
H	−0.542 873 140 823 97	−0.655 858 368 925 34	−1.511 638 064 717 59
H	0.197 915 933 636 86	0.718 581 113 619 93	−1.658 250 321 069 71

TABLE S3. Optimized Cartesian coordinates of the BLYP-D3 H_3OF structure.

atom	x [Å]	y [Å]	z [Å]
H	−0.166 985 377 522 74	−0.067 111 706 848 54	−0.155 623 334 494 68
O	−0.214 294 712 675 84	−0.332 358 023 776 12	0.781 827 350 161 14
H	0.817 080 681 135 79	0.442 182 808 071 18	1.583 202 191 824 64
H	−1.110 345 961 663 35	−0.074 171 186 399 60	1.067 619 068 498 40
F	1.501 667 370 726 12	0.929 701 108 953 07	2.113 400 724 010 48

TABLE S4. Optimized Cartesian coordinates of the BLYP-D3 H_3OCl structure.

atom	x [Å]	y [Å]	z [Å]
H	−0.150 584 288 800 15	−0.155 968 253 448 04	−0.145 361 223 972 40
O	−0.188 024 378 636 13	−0.382 733 479 003 34	0.803 952 580 176 78
H	0.733 552 634 288 05	0.555 163 545 389 02	1.519 866 733 036 24
H	−1.098 423 962 946 23	−0.160 063 074 911 49	1.078 673 469 271 57
Cl	1.616 813 240 139 49	1.402 206 228 361 45	2.200 313 114 704 66

TABLE S5. Optimized Cartesian coordinates of the BLYP-D3 $H_9O_4^+$ structure.

atom	x [Å]	y [Å]	z [Å]
O	−0.110 163 286 365 06	0.225 539 642 143 94	−0.100 706 177 316 48
H	−0.028 908 982 701 97	0.264 715 990 960 13	0.930 760 868 687 33
H	0.836 926 715 502 22	0.151 869 503 117 01	−0.511 764 022 980 53
H	−0.653 888 567 823 13	−0.618 657 144 090 80	−0.355 200 422 957 49
O	2.264 190 861 728 00	0.097 484 767 436 09	−1.113 239 732 680 60
H	2.565 575 866 738 45	−0.829 162 857 247 42	−1.155 171 064 865 15
H	2.242 962 334 210 16	0.393 555 530 189 20	−2.042 387 671 549 16
O	−1.469 884 782 547 27	−1.863 240 581 807 04	−0.771 501 198 921 74
H	−1.176 785 447 456 21	−2.630 634 769 380 02	−0.245 384 076 908 10
H	−2.408 103 330 821 26	−1.743 206 607 126 73	−0.533 161 364 238 92
O	0.009 343 636 775 46	0.320 657 157 865 44	2.477 837 366 437 62
H	0.705 445 522 334 30	−0.276 486 878 492 32	2.809 877 577 473 02
H	0.282 425 460 426 32	1.209 039 246 432 57	2.773 411 919 820 21

TABLE S6. Optimized Cartesian coordinates of the BLYP-D3 H_7O_3F structure.

atom	x [Å]	y [Å]	z [Å]
O	−0.283 756 167 495 59	0.574 990 958 688 39	−0.162 208 024 213 60
H	−0.128 453 836 874 77	0.413 736 143 530 71	0.809 501 806 148 06
H	0.606 456 093 253 24	0.450 924 670 929 13	−0.593 603 418 532 07
H	−1.172 555 373 888 74	−0.405 416 914 011 80	−0.650 610 446 791 61
O	2.179 528 432 845 69	0.208 167 408 362 34	−1.328 742 095 633 57
H	2.826 605 925 964 24	0.256 571 839 987 10	−0.601 312 765 020 36
H	2.210 785 149 408 70	−0.724 569 370 531 42	−1.609 494 668 660 01
F	−1.837 877 130 172 01	−1.103 790 099 729 76	−1.013 917 500 577 98
O	0.123 270 447 265 16	0.084 601 780 987 14	2.507 781 488 145 77
H	0.721 746 855 505 43	−0.683 621 584 053 56	2.546 512 279 744 75
H	0.648 440 437 108 61	0.813 096 672 982 87	2.886 132 309 744 41

TABLE S7. Optimized Cartesian coordinates of the BLYP-D3 H_7O_3Cl structure.

atom	x [Å]	y [Å]	z [Å]
O	−0.044 627 026 692 27	−0.824 039 219 100 89	−0.237 698 219 434 93
H	−0.086 784 509 951 58	−0.504 473 567 968 14	0.755 498 127 318 64
H	0.808 932 441 551 44	−0.420 761 647 802 06	−0.660 951 905 285 80
H	−0.874 826 335 930 19	−0.440 561 367 788 86	−0.721 389 834 122 12
O	2.101 366 041 921 49	0.154 435 679 498 68	−1.309 564 946 311 88
H	2.176 976 020 777 47	1.101 371 494 758 82	−1.088 124 893 161 98
H	2.883 821 490 360 02	−0.258 978 829 274 18	−0.899 318 362 253 62
O	−2.135 777 932 007 21	0.106 928 020 956 81	−1.450 735 614 964 15
H	−2.228 441 703 348 17	1.058 588 290 045 48	−1.257 753 669 818 08
H	−2.934 966 050 817 94	−0.301 884 545 087 21	−1.069 129 384 978 08
Cl	−0.176 432 435 863 05	0.085 579 691 761 54	2.536 004 703 012 02

TABLE S8. Optimized Cartesian coordinates of the BLYP-D3 $H_5O_2F_2^-$ structure.

atom	x [Å]	y [Å]	z [Å]
O	−0.275 681 509 616 49	0.523 376 438 886 68	−0.136 768 664 193 35
H	−0.072 785 711 083 08	0.322 978 943 051 06	0.906 041 034 889 69
H	0.578 275 531 986 54	0.379 271 247 333 72	−0.618 954 212 338 43
H	−1.132 306 363 866 51	−0.282 083 411 761 58	−0.590 838 827 739 04
O	2.154 134 831 920 55	0.084 219 201 695 62	−1.444 108 322 489 18
H	2.825 939 624 240 80	0.583 006 461 632 10	−0.944 821 059 812 66
H	2.365 057 239 200 45	−0.847 970 723 056 73	−1.254 544 654 613 80
F	−1.912 898 771 942 12	−0.972 215 653 954 83	−0.998 797 147 630 46
F	0.179 292 018 159 82	0.082 851 953 273 97	2.209 757 551 627 24

TABLE S9. Optimized Cartesian coordinates of the BLYP-D3 $H_5O_2Cl_2^-$ structure.

atom	x [Å]	y [Å]	z [Å]
O	0.108 566 725 978 14	−0.995 992 099 558 45	−0.033 870 143 776 64
H	0.003 756 970 148 17	−0.524 922 655 949 77	0.889 817 612 056 08
H	0.876 618 239 948 82	−0.522 458 825 782 04	−0.538 104 662 805 13
H	−0.769 175 908 385 16	−0.857 475 974 273 07	−0.579 370 426 762 45
O	2.029 140 535 289 28	0.171 250 716 713 28	−1.327 627 443 317 43
H	2.034 314 872 635 02	1.123 546 797 530 37	−1.116 830 529 796 57
H	2.892 559 299 889 74	−0.157 531 314 943 75	−1.015 254 747 500 54
Cl	−2.337 489 383 669 70	−0.645 577 797 664 37	−1.609 337 989 733 47
Cl	−0.128 331 878 734 32	0.356 675 600 827 83	2.555 550 584 036 16

TABLE S10. Optimized Cartesian coordinates of the DSD-PBEP86-D3 H_2O structure.

atom	x [Å]	y [Å]	z [Å]
O	0.000 000 000 000 00	−0.000 000 081 689 94	0.005 733 423 566 23
H	0.000 000 000 000 00	0.754 391 462 493 41	−0.589 946 744 942 21
H	0.000 000 000 000 00	−0.754 391 380 803 47	−0.589 946 678 624 02

TABLE S11. Optimized Cartesian coordinates of the DSD-PBEP86-D3 $H_5O_2^+$ structure.

atom	x [Å]	y [Å]	z [Å]
H	0.190 940 798 004 85	−0.001 221 854 873 13	−0.000 112 632 811 92
O	0.236 136 170 884 88	0.142 912 975 424 68	1.190 049 383 596 22
H	0.194 563 375 458 45	−0.712 048 458 121 09	1.642 677 122 896 41
H	−0.521 168 989 195 60	0.658 284 418 439 09	1.503 993 024 628 95
O	0.231 793 579 602 23	−0.146 148 648 042 01	−1.190 579 186 660 67
H	−0.533 249 462 958 29	−0.651 025 152 400 32	−1.502 793 464 122 70
H	0.200 984 528 203 50	0.709 246 719 572 78	−1.643 234 247 526 29

TABLE S12. Optimized Cartesian coordinates of the DSD-PBEP86-D3 H_3OF structure.

atom	x [Å]	y [Å]	z [Å]
H	−0.176 496 319 718 99	−0.077 223 831 210 88	−0.151 569 725 119 96
O	−0.214 721 355 673 48	−0.313 162 872 450 75	0.782 202 110 970 13
H	0.828 583 923 132 73	0.454 178 597 399 39	1.591 492 321 881 96
H	−1.109 955 650 335 60	−0.084 793 020 623 44	1.056 638 104 610 41
F	1.499 711 402 595 33	0.919 244 126 885 67	2.111 663 187 657 45

TABLE S13. Optimized Cartesian coordinates of the DSD-PBEP86-D3 H_3OCl structure.

atom	x [Å]	y [Å]	z [Å]
H	−0.172 372 078 052 34	−0.177 858 852 207 07	−0.149 435 087 767 23
O	−0.197 470 139 210 92	−0.368 736 666 714 46	0.796 043 315 053 96
H	0.764 263 357 952 41	0.591 406 512 696 66	1.541 794 282 964 05
H	−1.106 271 000 373 63	−0.180 840 376 005 34	1.060 039 720 763 03
Cl	1.625 183 103 729 50	1.394 634 348 617 81	2.209 002 442 203 05

TABLE S14. Optimized Cartesian coordinates of the DSD-PBEP86-D3 $H_9O_4^+$ structure.

atom	x [Å]	y [Å]	z [Å]
O	−0.075 645 591 926 74	0.163 578 644 039 42	−0.068 485 275 956 78
H	0.017 191 666 435 26	0.212 057 943 307 23	0.942 499 826 498 13
H	0.842 671 676 166 64	0.104 354 993 189 31	−0.498 655 054 934 42
H	−0.626 720 102 627 29	−0.653 376 340 062 28	−0.318 849 733 403 91
O	2.245 626 569 013 49	0.089 344 299 309 45	−1.135 513 771 484 16
H	2.601 524 972 967 63	−0.803 286 036 365 70	−1.211 594 603 058 49
H	2.239 970 838 607 74	0.430 999 414 974 92	−2.036 873 411 487 38
O	−1.471 057 111 105 99	−1.860 213 316 986 38	−0.752 845 156 822 13
H	−1.309 079 390 458 78	−2.633 328 944 464 10	−0.200 245 204 325 39
H	−2.416 793 162 327 86	−1.691 713 309 386 12	−0.671 859 730 203 29
O	0.061 498 895 269 22	0.286 985 839 398 52	2.477 855 883 265 34
H	0.845 749 369 281 21	−0.139 662 264 266 81	2.841 262 535 013 60
H	0.104 197 370 705 47	1.195 732 077 312 58	2.796 675 696 898 87

TABLE S15. Optimized Cartesian coordinates of the DSD-PBEP86-D3 H_7O_3F structure.

atom	x [Å]	y [Å]	z [Å]
O	−0.215 860 237 551 27	0.524 294 145 465 57	−0.143 785 184 853 08
H	−0.070 611 215 441 56	0.360 281 145 826 30	0.811 100 076 725 30
H	0.643 790 233 301 82	0.351 538 564 768 82	−0.580 826 428 376 74
H	−1.237 490 340 852 13	−0.342 213 412 481 85	−0.657 595 793 497 23
O	2.147 083 553 909 66	0.053 875 126 380 98	−1.441 986 372 108 36
H	2.845 476 284 629 56	0.634 097 863 197 27	−1.121 370 763 337 59
H	2.471 856 952 540 37	−0.832 455 093 314 08	−1.252 090 284 459 31
F	−1.958 677 074 428 26	−0.922 422 103 646 71	−1.019 313 507 840 08
O	0.083 458 733 029 71	0.046 438 220 822 11	2.532 833 955 451 90
H	0.621 785 086 147 83	−0.738 172 146 461 15	2.680 194 047 632 30
H	0.563 378 857 634 20	0.749 429 196 583 88	2.982 879 219 016 69

TABLE S16. Optimized Cartesian coordinates of the DSD-PBEP86-D3 H_7O_3Cl structure.

atom	x [Å]	y [Å]	z [Å]
O	−0.048 345 222 733 17	−0.783 575 713 174 50	−0.219 062 842 169 51
H	−0.086 132 546 364 49	−0.492 357 247 383 44	0.756 786 739 294 21
H	0.795 373 278 273 91	−0.398 628 785 925 22	−0.636 719 328 234 18
H	−0.866 901 469 283 60	−0.411 797 608 108 77	−0.695 191 484 658 88
O	2.080 166 657 811 74	0.145 490 958 844 76	−1.292 841 528 907 35
H	2.195 025 537 561 92	1.086 301 007 626 55	−1.115 910 789 426 79
H	2.870 177 969 576 79	−0.271 883 325 141 89	−0.930 744 233 711 43
O	−2.111 665 278 353 09	0.110 950 415 023 56	−1.435 994 636 670 36
H	−2.258 872 269 109 72	1.047 786 111 796 63	−1.262 011 989 404 33
H	−2.916 409 225 976 18	−0.325 732 173 301 11	−1.133 854 907 034 82
Cl	−0.163 177 431 404 09	0.049 650 359 743 42	2.562 381 000 923 47

TABLE S17. Optimized Cartesian coordinates of the DSD-PBEP86-D3 $H_5O_2F_2^-$ structure.

atom	x [Å]	y [Å]	z [Å]
O	−0.100 928 565 515 95	0.397 499 014 920 49	−0.175 517 291 350 83
H	−0.045 320 323 423 95	0.405 094 162 136 89	0.797 480 579 105 31
H	0.871 273 928 444 66	0.256 195 960 792 41	−0.525 054 958 057 13
H	−0.911 877 838 828 59	−0.578 214 229 787 06	−0.569 983 950 829 08
F	2.153 918 075 423 77	0.076 724 320 687 68	−0.999 051 474 828 47
H	0.578 674 022 718 97	−0.371 540 424 181 36	2.879 366 022 511 12
H	0.568 112 740 899 71	1.144 949 867 614 47	2.918 748 198 531 70
F	−1.560 751 305 045 05	−1.324 082 197 342 63	−0.892 989 887 318 07
O	0.054 824 248 226 44	0.390 576 314 059 10	2.611 402 477 335 44

TABLE S18. Optimized Cartesian coordinates of the DSD-PBEP86-D3 $H_5O_2Cl_2^-$ structure.

atom	*x* [Å]	*y* [Å]	*z* [Å]
O	0.119 962 356 160 92	−0.941 868 497 652 33	−0.004 080 424 024 72
H	−0.030 699 530 036 91	−0.482 491 353 385 52	0.893 374 192 485 53
H	0.866 921 124 657 04	−0.455 026 701 710 1	−0.494 283 453 448 53
H	−0.733 113 749 447 69	−0.865 345 350 817 08	−0.557 311 316 779 56
O	1.984 472 724 643 96	0.248 691 161 981 6	−1.290 038 004 106 44
H	2.067 304 030 975 27	1.176 748 561 032 77	−1.042 321 756 322 05
H	2.853 002 936 418 95	−0.132 262 511 656 43	−1.116 784 754 918
Cl	−2.296 011 353 383 2	−0.753 326 658 680 51	−1.613 305 014 836 88
Cl	−0.271 325 539 988 34	0.355 550 350 887 6	2.570 813 531 950 65

TABLE S19. Optimized Cartesian coordinates of the MP2 H_2O structure.

atom	*x* [Å]	*y* [Å]	*z* [Å]
O	0.000 000 000 000 00	0.000 000 000 661 33	0.007 652 889 219 56
H	0.000 000 000 000 00	0.752 324 818 189 08	−0.590 906 445 150 89
H	0.000 000 000 000 00	−0.752 324 818 850 41	−0.590 906 444 068 67

TABLE S20. Optimized Cartesian coordinates of the MP2 $H_5O_2^+$ structure.

atom	*x* [Å]	*y* [Å]	*z* [Å]
H	0.193 548 195 993 18	−0.001 123 119 462 03	−0.000 069 062 582 08
O	0.239 510 926 250 67	0.145 300 184 572 04	1.189 041 615 546 20
H	0.193 200 571 053 68	−0.711 493 262 536 64	1.639 561 934 194 82
H	−0.524 550 121 949 12	0.654 748 844 298 24	1.498 841 442 981 60
O	0.235 173 610 878 21	−0.148 535 784 500 77	−1.189 545 861 938 26
H	−0.536 556 377 303 50	−0.647 476 678 744 77	−1.497 420 971 107 56
H	0.199 673 195 076 91	0.708 579 816 373 94	−1.640 409 097 094 72

TABLE S21. Optimized Cartesian coordinates of the MP2 H_3OF structure.

atom	x [Å]	y [Å]	z [Å]
H	−0.177 526 642 434 35	−0.076 020 965 262 88	−0.150 291 019 900 65
O	−0.212 836 421 143 28	−0.314 872 831 428 43	0.783 483 178 078 33
H	0.827 402 518 820 24	0.452 919 338 422 79	1.590 497 234 992 50
H	−1.108 616 284 680 19	−0.083 317 083 986 70	1.055 603 836 622 91
F	1.498 698 829 437 58	0.919 534 542 255 20	2.111 132 770 206 91

TABLE S22. Optimized Cartesian coordinates of the MP2 H_3OCl structure.

atom	x [Å]	y [Å]	z [Å]
H	−0.174 032 778 404 85	−0.177 522 151 587 54	−0.148 685 957 470 86
O	−0.195 603 943 091 50	−0.368 233 779 730 86	0.797 684 494 404 09
H	0.765 869 075 520 56	0.592 375 897 004 79	1.543 539 678 501 71
H	−1.106 498 618 360 79	−0.181 110 919 178 83	1.057 729 682 483 53
Cl	1.623 599 508 381 60	1.393 095 919 880 04	2.207 176 775 298 39

TABLE S23. Optimized Cartesian coordinates of the MP2 $H_9O_4^+$ structure.

atom	x [Å]	y [Å]	z [Å]
O	−0.080 043 685 278 78	0.171 175 040 125 99	−0.067 253 351 656 36
H	0.012 933 683 269 55	0.216 161 777 739 96	0.944 198 067 973 08
H	0.840 139 114 355 55	0.105 982 825 901 56	−0.493 449 108 168 88
H	−0.627 875 762 295 05	−0.648 714 304 587 17	−0.317 118 212 833 22
O	2.241 034 932 372 43	0.084 224 100 459 34	−1.129 868 747 836 48
H	2.588 167 536 047 23	−0.811 836 922 134 40	−1.214 117 093 914 72
H	2.232 938 232 805 77	0.424 937 772 281 68	−2.032 303 956 378 66
O	−1.462 239 284 532 56	−1.855 547 275 484 94	−0.758 720 499 555 39
H	−1.292 887 076 686 40	−2.633 112 981 350 08	−0.213 463 360 468 99
H	−2.409 753 691 629 71	−1.698 854 236 819 94	−0.667 587 707 885 46
O	0.057 770 995 737 41	0.291 807 232 954 82	2.477 547 093 376 22
H	0.836 963 542 205 68	−0.145 418 004 856 57	2.841 073 936 943 57
H	0.121 987 463 628 86	1.200 667 975 769 79	2.794 434 940 405 28

TABLE S24. Optimized Cartesian coordinates of the MP2 H_7O_3F structure.

atom	x [Å]	y [Å]	z [Å]
O	−0.220 046 692 787 72	0.524 666 153 215 51	−0.145 527 505 379 34
H	−0.071 970 683 742 16	0.359 101 401 243 24	0.808 968 068 181 14
H	0.641 089 091 698 90	0.352 360 600 548 72	−0.580 524 607 920 64
H	−1.236 359 422 148 45	−0.340 225 488 293 82	−0.657 875 811 834 01
O	2.144 724 772 672 37	0.053 435 056 680 62	−1.440 499 477 436 32
H	2.845 364 557 330 06	0.632 051 820 348 11	−1.120 209 526 751 14
H	2.473 473 976 256 57	−0.831 306 772 847 43	−1.247 351 986 801 13
F	−1.957 848 099 747 82	−0.922 405 848 187 69	−1.020 516 703 203 43
O	0.083 708 515 352 77	0.046 399 673 090 40	2.531 088 595 323 49
H	0.625 145 975 060 88	−0.736 465 683 398 65	2.680 082 584 851 01
H	0.566 908 842 974 54	0.747 080 594 742 13	2.982 405 335 324 17

TABLE S25. Optimized Cartesian coordinates of the MP2 H_7O_3Cl structure.

atom	x [Å]	y [Å]	z [Å]
O	−0.047 978 268 091 81	−0.792 717 980 264 14	−0.221 453 801 686 80
H	−0.086 309 640 361 33	−0.493 533 814 902 14	0.753 170 698 149 26
H	0.794 370 978 648 12	−0.402 162 473 109 67	−0.638 032 216 324 50
H	−0.865 328 760 257 75	−0.415 881 764 597 51	−0.696 710 323 814 37
O	2.075 456 625 777 65	0.146 533 491 161 90	−1.291 449 509 246 11
H	2.190 896 167 452 39	1.086 661 517 722 45	−1.107 733 506 157 28
H	2.867 398 952 635 89	−0.267 911 067 523 68	−0.928 338 544 023 11
O	−2.107 681 176 098 32	0.110 582 385 068 02	−1.434 355 374 493 41
H	−2.253 724 085 385 85	1.047 667 849 223 17	−1.257 019 838 313 93
H	−2.914 360 665 875 30	−0.320 552 404 681 77	−1.127 303 045 953 99
Cl	−0.163 500 128 443 67	0.057 518 261 903 36	2.546 061 461 864 25

TABLE S26. Optimized Cartesian coordinates of the MP2 $H_5O_2F_2^-$ structure.

atom	x [Å]	y [Å]	z [Å]
O	−0.104 379 622 298 73	0.401 099 698 656 54	−0.180 418 957 775 05
H	−0.047 369 684 966 74	0.405 430 053 330 87	0.793 160 662 485 25
H	0.871 439 913 930 22	0.255 840 294 308 88	−0.527 227 975 663 55
H	−0.903 263 792 901 98	−0.570 560 572 160 15	−0.563 881 028 113 23
F	2.147 659 662 883 66	0.074 282 886 980 48	−0.994 186 622 291 54
H	0.576 416 519 203 38	−0.372 814 971 856 82	2.876 698 148 377 06
H	0.569 183 450 346 24	1.140 275 769 408 50	2.918 201 686 554 13
F	−1.554 857 224 338 26	−1.325 607 099 823 88	−0.883 712 744 802 09
O	0.053 095 761 042 22	0.389 256 730 055 56	2.605 766 546 329 02

TABLE S27. Optimized Cartesian coordinates of the MP2 $H_5O_2Cl_2^-$ structure.

atom	x [Å]	y [Å]	z [Å]
O	0.122 670 380 992 15	−0.952 995 697 125 91	−0.003 553 923 071 07
H	−0.028 690 637 136 68	−0.485 067 363 826 37	0.890 939 897 039 71
H	0.867 139 534 566 17	−0.461 212 206 296 85	−0.494 221 501 590 96
H	−0.730 992 243 790 19	−0.867 382 271 577 59	−0.557 014 650 993 11
O	1.978 156 353 607 51	0.247 976 117 841 73	−1.288 902 032 151 15
H	2.059 291 433 012 48	1.175 526 981 864 63	−1.035 976 055 243 49
H	2.849 740 116 873 14	−0.126 634 136 658 11	−1.113 211 688 849 14
Cl	−2.286 875 190 236 99	−0.741 227 770 715 67	−1.604 102 484 568 60
Cl	−0.269 926 747 887 59	0.361 685 346 494 14	2.552 105 439 427 81

TABLE S28. Optimized Cartesian coordinates of the CCSD(T) H_2O structure.

atom	x [Å]	y [Å]	z [Å]
O	0.000 000 000 000 00	0.000 000 003 888 30	0.007 233 911 530 94
H	0.000 000 000 000 00	0.752 775 661 123 57	−0.590 696 955 089 98
H	0.000 000 000 000 00	−0.752 775 665 011 87	−0.590 696 956 440 96

TABLE S29. Optimized Cartesian coordinates of the CCSD(T) $H_5O_2^+$ structure.

atom	x [Å]	y [Å]	z [Å]
H	0.193 126 969 263 05	−0.001 250 986 997 13	−0.000 086 613 768 54
O	0.238 584 231 381 02	0.145 208 653 021 91	1.187 943 149 287 45
H	0.194 884 060 751 07	−0.710 784 264 764 48	1.638 842 212 535 57
H	−0.525 059 881 153 99	0.654 000 179 190 93	1.497 835 438 522 49
O	0.234 223 225 210 33	−0.148 452 089 119 50	−1.188 484 621 638 53
H	−0.537 063 319 718 74	−0.646 690 682 996 70	−1.496 580 092 124 27
H	0.201 304 714 267 28	0.707 969 191 664 99	−1.639 469 472 814 17

TABLE S30. Optimized Cartesian coordinates of the CCSD(T) H_3OF structure.

atom	x [Å]	y [Å]	z [Å]
H	−0.179 621 207 233 81	−0.076 482 137 082 41	−0.151 800 096 809 41
O	−0.216 422 391 848 46	−0.318 501 003 556 03	0.780 615 706 558 65
H	0.833 101 159 835 58	0.456 314 938 289 11	1.595 029 855 830 42
H	−1.110 454 288 482 75	−0.083 696 518 822 84	1.054 040 994 052 26
F	1.500 518 727 729 43	0.920 607 721 172 17	2.112 539 540 368 07

TABLE S31. Optimized Cartesian coordinates of the CCSD(T) H_3OCl structure.

atom	x [Å]	y [Å]	z [Å]
H	−0.195 015 024 649 66	−0.191 530 626 944 03	−0.162 924 386 458 82
O	−0.216 610 159 728 87	−0.386 758 914 210 99	0.781 263 008 515 17
H	0.801 134 126 669 15	0.622 029 286 747 08	1.571 233 008 576 90
H	−1.125 316 038 072 57	−0.194 998 981 461 80	1.041 043 857 549 66
Cl	1.649 140 339 826 96	1.409 864 202 257 34	2.226 829 185 033 96
